# Gender and ideological orientation moderate the influence of climate misinformation on pro‐environmental behavioural intentions

**DOI:** 10.1111/bjso.70000

**Published:** 2025-07-01

**Authors:** Aitor Larzabal‐Fernandez, Angela Castrechini Trotta, Alexandra Vázquez

**Affiliations:** ^1^ Departamento Psicología Evolutiva y de la Educación Universidad del País Vasco (UPV/EHU) Leioa Spain; ^2^ Departamento de Psicología Social y de las Organizaciones Universitat de Barcelona (UB) Barcelona Spain; ^3^ Departamento de Psicología Social y Psicología Cuantitativa Universidad Nacional de Educación a Distancia (UNED) Madrid Spain

**Keywords:** attitudes towards climate change, climate misinformation, gender, ideological orientation, pro‐environmental behaviour

## Abstract

Climate change is a significant and urgent challenge faced by humanity, yet the widespread dissemination of misinformation hampers progress in combating it. While previous research shows that false information about the scientific consensus on climate change can shape beliefs and attitudes, its effect on behavioural intentions remains less understood. To examine this, two experiments in Spain (*n* = 673) and Ecuador (*n* = 365) tested the impact of denialist versus confirmatory or neutral messages about the scientific consensus on anthropogenic climate change on the intention to take pro‐environmental actions. Moreover, we explored the moderating roles of gender and ideological orientation, which are key factors in climate scepticism. In both countries, right‐wing men who received consensus‐denying messages showed fewer intentions to perform pro‐environmental behaviours compared to those who received consensus‐confirming messages. Consensus misinformation did not appear to have a consistent impact on women across ideological lines or on left‐wing men. These findings highlight the urgent need to develop communication interventions targeted at specific demographic subgroups to counteract climate misinformation and promote pro‐environmental actions.

## INTRODUCTION

Misinformation about climate change poses a significant threat to global efforts to mitigate its effects and has emerged as one of the primary obstacles to effective climate action (Imundo & Rapp, 2022; Lewandowsky & van der Linden, 2021). The United Nations categorizes climate change as enduring alterations in temperature and weather patterns. They assert that since the 19th century, these changes have predominantly been driven by human activities, particularly the burning of fossil fuels (United Nations, 2023). The Intergovernmental Panel on Climate Change (IPCC) states that human activities, such as burning fossil fuels and deforestation, are the main cause of greenhouse gas emissions and global warming (IPCC, 2023). Presently, climate change stands as one of the most pressing challenges confronting humanity. Even the most sanguine predictions of potential changes paint a bleak picture of life as we understand it (IPCC, 2019).

The spread of misinformation about climate change has become a major obstacle to climate action, eroding trust in science and polarizing public opinion (Van der Linden, 2021). In fact, while scientific consensus was once the primary barrier, climate misinformation is now considered a key impediment to effective action (Ulrich, 2022). Although misinformation about climate change can take various forms (e.g. denying its existence, downplaying its impacts), we focus here on the misrepresentation of the scientific consensus regarding the anthropogenic origins of climate change. While there is extensive experimental research on the effects of communicating scientific consensus, less is known about the impact of denialist messages regarding this consensus, particularly on behavioural intentions. Therefore, we will examine whether exposure to denialist messages about scientific consensus (as opposed to affirming or neutral messages) influences individuals' willingness to engage in pro‐environmental behaviour and related factors. Additionally, we will investigate the potential moderating role of the interaction between gender and ideological orientation, given previous evidence suggesting relatively higher levels of scepticism among right‐wing men (McCright & Dunlap, 2011; Poortinga et al., 2019).

### The impact of communicating scientific consensus

Mass media notably influences the discourse and public understanding of climate change (Boykoff, 2008). As platforms for constructing and consolidating social representations of environmental issues, both traditional and social media play a crucial part (Castrechini, 2023; Pol et al., 2024). Various studies propose that the proliferation of misinformation through blogs, social networks and conservative think tanks complicates the climate change debate, escalates political polarization (Treen et al., 2020) and obstructs mitigation policies (Abudu et al., 2023).

There are four main types of climate change misinformation: denial of climate change existence, denial of human‐attributed causes, denial of its impact (Björnberg et al., 2017) and denial of scientific consensus (McCright, 2016). Research shows that the denial of climate change existence is less common, except in the United States, while the other three types prevail (Koehler, 2016). The current research focuses on the impact of the denial of scientific consensus on the anthropogenic origins of climate change on intentions to engage in pro‐environmental actions.

Communication about scientific consensus can have a strong influence, as it might serve as a ‘gateway’ to shaping other beliefs and encouraging support for climate change mitigation policies (Van der Linden et al., 2015). A substantial body of existing literature indicates that messages affirming scientific consensus tend to enhance beliefs in such consensus (Bolsen & Druckman, 2018; Chinn & Hart, 2021; Deryugina & Shurchkov, 2016; Dixon et al., 2017; Tschötschel et al., 2021; Van Stekelenburg et al., 2022). For instance, a study across 27 countries (*n* = 10,527) found that communicating the scientific consensus on human‐caused climate change (compared to a neutral topic) significantly reduced climate change misperceptions and slightly increased climate worry, particularly among right‐leaning individuals, although it had no effect on support for public action (Većkalov et al., 2024). Additionally, a pre‐registered meta‐analysis revealed that a single exposure to consensus messaging had a small, but positive effect on perceived scientific consensus on climate change and on beliefs in scientific facts (Van Stekelenburg et al., 2022).

While communicating scientific consensus seems to be an effective way to change perceptions of consensus and beliefs about contested science topics, the effect of consensus messages on support for climate‐related behaviours or policies remains unclear (Bayes et al., 2020). Different studies on communicating scientific consensus have not found direct effects on policy support, preferences or intended actions (e.g. Bolsen & Druckman, 2018; Deryugina & Shurchkov, 2016; Tschötschel et al., 2021). However, Bolsen and Druckman's (2018) findings suggest that consensus messages may indirectly influence support for climate mitigation policies by strengthening beliefs in human‐induced climate change.

### The impact of denying scientific consensus

Interventions aimed at fostering scepticism, through the spread of various types of misinformation—not just consensus—shows a significantly stronger average effect on attitudes compared to those designed to promote belief in climate change (Rode et al., 2021). Given that belief in climate change appears to be more easily undermined than reinforced, it is crucial to understand the consequences of climate misinformation and the factors that amplify its effects. However, while numerous experiments have examined the effects of communicating scientific consensus, there is less causal evidence about the consequences of denying such consensus.

Most of the research on the impact of denialist messages has been conducted within the framework of inoculation theory, with the goal of mitigating the adverse effects of misinformation (Cook, Cook, 2019; Cook et al., 2017; Spampatti et al., 2024). These studies show that denialist messages about scientific consensus can indeed undermine the perception of that consensus (Maertens et al., 2020; Van der Linden et al., 2017). Additionally, Cook et al. (2017) demonstrated that misinformation about scientific consensus induced a polarizing effect on climate attitudes: individuals with low support for free‐market principles became more accepting of climate acceptance, while those with strong free‐market beliefs became less accepting. This suggests that certain ideologies can moderate reactions to false consensus messages. However, support for free‐market economics may hold particular salience within the US context and may not translate as directly to other cultural or political settings. Furthermore, Cook et al. did not analyse the potential moderating role of gender, and their focus remained on perceptions and attitudes (perceived scientific consensus, acceptance of anthropogenic global warming, attribution, trust in climate scientists and policy support) rather than on behavioural intentions.

Indeed, current evidence remains insufficient to conclusively determine whether exposure to messages about the existence or absence of scientific consensus influences intentions to engage in pro‐environmental actions to mitigate climate change (Bayes et al., 2020; McCright et al., 2016). Additionally, most studies have been conducted in the United States and the United Kingdom (Herasimenka et al., 2025), which may limit the generalizability of these findings across diverse cultural, economic and social contexts. This work aims to enhance our understanding of how messages that deny (vs. affirm) scientific consensus impact behavioural intentions depending on individuals' ideological orientation and gender across two distinct cultural contexts, one of which is non‐WEIRD.

### Potential moderation effects of gender and ideological orientation

Previous research shows that climate scepticism is greater among individuals with right‐wing political ideologies (Berkebile‐Weinberg et al., 2024; Hornsey et al., 2018; Huber, 2020) and among men (McCright & Dunlap, 2011; Poortinga et al., 2019; Vázquez et al., 2021). Right‐wing men, in particular, appear more likely than other demographic groups to endorse denialist beliefs about climate change (McCright & Dunlap, 2011). Indeed, there has been a growing convergence between far‐right and climate denial groups, emphasizing anti‐establishment rhetoric, promotion of doubt, industrial or supplier masculinities and nationalism (Hultman et al., 2019).

We propose that recipients' reaction to denialist messages might partly depend on their political alignment and gender. Some research indicates that messages validating scientific consensus may trigger defiance among conservative individuals and those who deny human‐induced climate change. Climate change denial may be motivated by a preference for the status quo (Jost et al., 2004) and resistance to change (Lewandowsky et al., 2012), especially if the cultural and economic changes resulting from climate‐oriented reforms are perceived as a threat to personal values or interests (Feygina et al., 2010). Resistance to change may be particularly strong among those who benefit most from the existing social and economic order (Jost et al., 2004). For conservative men, accepting the reality of climate change and the need for action might be perceived as a threat to their group identity, particularly if climate action is framed as being in conflict with traditional values or economic interests (Feygina et al., 2010).

Additionally, online sharing of climate change information is often polarized along ideological lines, with distinct groups sharing information from different sources, reinforcing existing beliefs (Cann et al., 2021). Right‐wing media commonly disseminate misinformation about climate change (Björnberg et al., 2017). Consequently, conservative individuals, who frequently consume such media, might process information affirming a lack of consensus more readily than liberal individuals. This may lead to stronger credibility being ascribed to denialist messages among conservative participants compared to liberal participants (Hertwig et al., 2008). Particularly, conservative men, known for their heightened climate scepticism compared to conservative women and liberal individuals, may most readily accept these views (Chinn & Hart, 2023; McCright & Dunlap, 2011) and use them as a justification to reduce their pro‐environmental behaviour.

### Overview of the current research

Despite the growing body of research on climate misinformation, few experimental studies have examined how the interaction between gender and ideological orientation moderates the influence of this type of message on behavioural intentions, especially in non‐Western contexts. We address this gap in knowledge by investigating the effects of consensus‐denying messages on the pro‐environmental intentions of men and women of different ideological orientations in Spain and Ecuador.

We conducted a pilot and two experimental studies. The pilot study was correlational and aimed at examining if the gender and ideological orientation of the participants affected their intention to perform pro‐environmental behaviours as well as other correlates such as attitudes towards these behaviours, perceived ability to control such behaviours, and their readiness to participate in collective action against climate change. Following this, two experimental studies were conducted to assess the impact of receiving messages that deny (vs. confirm) the existence of a scientific consensus on the human‐driven origin of climate change and whether recipients' gender and ideological orientation moderated this effect. The second experiment included a control condition where no information about the scientific consensus was provided.

We hypothesize that male participants with right‐wing political beliefs will show fewer intentions to engage in pro‐environmental behaviour, less positive attitudes towards such behaviour, less perceived control over these behaviours, and less collective action intentions when exposed to information that negates (vs confirms or keeps silent about) the existence of a scientific consensus around climate change. Statistically, these patterns of susceptibility to manipulation are expected to manifest as a three‐way interaction between the experimental condition, gender and ideological orientation across all the dependent variables.

### Open practices

In the interest of enhancing the reproducibility and transparency of our research, we have adhered to the principles of open science in several ways. All data, materials and detailed protocols used in our studies are freely available to other researchers and the public at https://osf.io/erjby/?view_only=0e1f7927bc624ae6854b03100362e396. The hypotheses, methods and analysis plans for our second study were pre‐registered prior to the commencement of data collection. This pre‐registration included detailed descriptions of the experimental design, expected data analyses and anticipated outcomes. The pre‐registration can be accessed at https://osf.io/th7pc?view_only=4b3bd36be32940da8a5c3a01bbe9766f.

## PILOT STUDY

Before conducting the experimental studies, we conducted a pilot study to address two key preliminary questions. First, we sought to assess the reliability and validity of the scale of pro‐environmental behavioural intentions, which was developed ad hoc and served as our main dependent variable. Second, we aimed to explore the influence of ideological orientation and gender on these intentions and on potential related variables such as climate change scepticism, attitudes towards pro‐environmental behaviours, perceived control over those actions and willingness to participate in pro‐environmental collective action. We hypothesized that individuals with right‐leaning political affiliations (as opposed to left‐leaning) and men (as opposed to women) would exhibit stronger climate change scepticism. Furthermore, we expected them to show less intention to engage in both individual and collective environmental actions, less positive attitudes towards these activities, and a lower sense of control regarding these behaviours.

### Method

#### Participants

A total of 406 (52% women; *M*_age_ = 34.67, *SD*_age_ = 14.94, range = 17–85 years) Spaniards were recruited for the study. A sensitivity analysis using G* Power (Erdfelder et al., 2009) revealed that the sample allowed the identification of a small effect size of *r* = .14, assuming a .05 significance level and 90% power. Data were collected using a snowball technique, in which distant learning Psychology students from a Spanish university encouraged their acquaintances to take part in an online study. These students, scattered across rural and urban regions of Spain, are typically older than regular university students and frequently juggle their studies with a professional career. Therefore, the resulting sample is more sociodemographically varied than what is typically obtained from university students. Participation was voluntary and without any compensation, but the participants were aware that the students would earn academic credit for their participation.

#### Procedure

Participants were invited to participate in an environmental issues survey via Qualtrics. Unless stated otherwise, the scales ranged from 0 (strongly disagree) to 6 (strongly agree). The survey incorporated the following scales:

##### Climate scepticism

We used the 12‐item scale of Climate Change Scepticism (Whitmarsh, 2011), which includes items such as ‘Claims that human activities are changing the climate are exaggerated’, *α* = .89. Prior psychometric analysis confirmed its cross‐gender equivalence (Larzabal‐Fernandez et al., 2024).

##### Intention to perform pro‐environmental behaviours

Based on a meta‐analysis that identifies the most effective methods to lower the carbon footprint (Ivanova et al., 2020), we developed a tailored scale to gauge the willingness to undertake 15 carbon footprint‐reducing behaviours. The scale ranged from 0 (Absolutely No) to 6 (Absolutely Yes). We focused on three categories of behaviours—transportation, diet and consumption/recycling—because they represent major areas of individual impact on carbon emissions, as identified by Ivanova et al. (2020). The factor analysis (see Supporting Information for more details) yielded three components: behaviours related to transportation (‘Living without a car’, *α* = .73), behaviours related to diet (‘Eating organic food’, *α* = .81) and behaviours related to reducing consumption and recycling (‘Buy fewer clothes’, *α* = .84). The analyses within the manuscript consider the full scale, while the Supporting Information contain separate analyses for each subcategory.

##### Attitudes towards pro‐environmental Behaviours

We developed a 3‐item scale based on the theory of planned behaviour (Lluna‐Ruiz et al., 2020; Palacios et al., 2014; Sarmiento et al., 2011): ‘I think it is important to carry out behaviors that reduce the carbon footprint’, ‘I am interested in knowing about behaviours that could serve to reduce the carbon footprint’ and ‘I enjoy performing behaviors that reduce the carbon footprint’, α = .74.

##### Perceived behavioural control over pro‐environmental behaviours

We used three items: (1) ‘Changing my behavior could have a positive and noticeable effect on climate change’, (2) ‘For me, performing pro‐environmental behaviors regularly over the next year would be…’ rated on a scale from 0 (extremely difficult) to 6 (extremely easy) and (3) ‘I feel that I am able to perform pro‐environmental behaviors on a regular basis over the next year’, rated from 0 (*very probably not*) to 6 (definitely yes). As all items were strongly interrelated, we computed a single indicator, *α* = .79.

##### Willingness to participate in collective action against climate change

We assessed participants' readiness to participate in seven different actions aimed at mitigating climate change, such as ‘signing a petition’ (based on Vázquez et al., 2021). This scale ranged from 0 (Not at all willing) to 6 (Completely willing), *α* = .89.

At the end of the questionnaire, we gathered information about participants' gender, age and ideological orientation. The latter was evaluated using two items, ranging from 1 (extreme left) to 7 (extreme right). Participants were asked to indicate their political leanings on economic issues (such as social welfare, government expenditure, taxation) and on social matters (including immigration, same‐gender marriage and abortion). A high correlation was found between the two questions, *r*_(404)_ = .67, *p* < .001, so the average of both responses was calculated to create a single measure of ideological orientation. Upon completing the questionnaire, all participants were debriefed and thanked.

### Results

#### Correlational analysis

Table 1 presents descriptive statistics and correlations for all variables. Right‐wing ideological orientation and being a man were found to be linked with increased scepticism towards climate change, lower intentions to engage in pro‐environmental behaviours, less positive attitudes towards such actions, less perceived control over them and diminished willingness to participate in collective action against climate change.

**TABLE 1:** Descriptive statistics and correlation matrix.

	*M*	*SD*	1	2	3	4	5	6
1. Ideological orientation	3.60	1.33						
2. Climate scepticism	2.45	1.28	.46*					
3. Behavioural intentions	4.77	1.01	−.36*	−.48*				
4. Attitudes	4.85	1.06	−.29*	−.44*	.59*			
5. Behavioural control	4.37	1.77	−.06	−.21*	.25*	.31*		
6. Collective action	4.06	1.52	−.44*	−.47*	.62*	.54*	.20*	
7. Gender			−.15*	−.18*	.19*	.22*	.13*	.19*

* The correlation is significant at the <.01 level; *n =* 406. Correlations with gender (0 women, 1 men) are point‐biserial coefficients.

The correlations between intention to perform pro‐environmental behaviours, attitudes and perceived control towards such behaviours, and collective action were positive. Conversely, scepticism showed a negative correlation with these factors.

Further correlation analyses, subdivided by gender, revealed slight differences in the link between variables for men and women (see Supporting Information).

#### Mean differences between men and women

Table 2 illustrates key differences between men and women across all variables. Men displayed greater scepticism towards climate change compared to women, reduced inclination to perform pro‐environmental behaviours, less favourable attitudes towards these actions, lower perceived control over these behaviours and diminished intentions to participate in collective action against climate change.

**TABLE 2:** Gender differences in climate scepticism, intention to perform pro‐environmental behaviours, attitudes towards pro‐environmental behaviours, perceived behavioural control over pro‐environmental behaviours and willingness to participate in collective action against climate change.

Variable	Men (*n* = 195)		Women (*n* = 211)		*t*	*p*	95% CI	
	*M*	*SD*	*M*	*SD*			Lower	Upper
Climate scepticism	2.69	1.40	2.19	1.08	4.00	<.001	0.25	0.73
Behavioural intentions	4.69	1.06	5.08	0.88	−4.05	<.001	−0.57	−0.19
Attitudes	4.63	1.15	5.10	0.88	−4.64	<.001	−0.67	−0.27
Behavioural control	4.08	1.84	4.60	1.65	−3.02	.003	−0.86	−.018
Collective action	3.72	1.60	4.33	1.38	−4.13	<.001	−0.90	−0.32

A regression analysis was conducted with ideological orientation, gender and their interaction as predictors and the remaining variables as outcomes. We found no significant interaction effects (see Supporting Information).

### Discussion

The pilot study's results, which aligned with previous studies, showed an association between right‐wing orientation and weaker intentions to perform pro‐environmental behaviours. Also consistently with previous evidence, men exhibited less willingness to perform pro‐environmental behaviours than women. This pattern was replicated across different variables. Although the interaction effects of ideological orientation and gender on the evaluated variables did not show significance, certain associations exhibited stronger ties in men than in women (see Supporting Information). Subsequent studies investigated whether the interaction between participants' ideological orientation and gender can moderate reactions to messages about the non‐existence of scientific consensus.

## STUDY 1: EFFECTS OF CONSENSUS‐DENYING MESSAGES ON PRO‐ENVIRONMENTAL INTENTIONS

Study 1 aimed to examine the impact of messages denying (vs. confirming) scientific consensus on the intention to perform pro‐environmental behaviours. It also explored attitudes and perceived control over these actions and willingness to participate in collective action against climate change considering participants' gender and ideological orientation as potential moderating factors. We hypothesized a significant three‐way interaction effect between the experimental manipulation, gender and ideological orientation on the dependent variables. More specifically, we anticipated that right‐wing men exposed to denialist messages would show less intentions to perform pro‐environmental behaviours, perceive less control over such actions, harbour less positive attitudes towards them, and be less willing to participate in collective action against climate change than right‐wing men informed about the existence of a scientific consensus. We did not expect significant effects between conditions among the remaining groups (left‐wing women, left‐wing men and right‐wing women).

### Method

#### Participants

We recruited 673 Spaniards (56.6% women, *M*_age_ = 33.69, *SD*_age_ = 13.30, age range = 17–81 years) using the snowball technique from the pilot study. Of the initial 921 participants, 248 were removed due to these reasons: they either exceeded or did not meet the set time limit of less than 500 s (*n* = 46) or more than 10,000 s (*n* = 65), were of a nationality other than Spanish (*n* = 71) or responded inaccurately to the attention control measure (*n* = 66).

To estimate the statistical power to detect the three‐way interaction between condition, gender and ideological orientation, we conducted a post‐hoc power analysis using linear regression models in R. A reduced model excluding the target interaction and a full model including it were compared to calculate the increase in explained variance (Δ*R* ^2^) associated with the addition of the three‐way term. This change in *R* ^2^ was used to compute the effect size (*f* ^2^), which was then entered into the pwr.f2.test function to estimate statistical power. Given the observed effect size and the sample size, the analysis revealed an estimated power of 0.906 to detect the Condition × Gender × Ideological Orientation interaction. This suggests that the study was well powered to detect this three‐way interaction. The code for these analyses in Studies 1 and 2 is available at https://osf.io/erjby/?view_only=0e1f7927bc624ae6854b03100362e396.

#### Procedure

Participants were invited to collaborate in an online study concerning environmental issues. The study commenced with a brief message about climate change that functioned as an experimental manipulation. In the *denialist condition*, it was indicated that ‘Some scientists claim that climate change is generated by human activity (…) But not all scientists agree with that, and there are many who think that climate change is generated by the sun’, which is one of the classic arguments used by climate change sceptics. In the scientific *consensus condition*, it was highlighted: ‘There is a broad consensus among scientists, which reaches 97%, that climate change is generated by human activity’. After that, we included an attention check: ‘According to the text we have shown you, is there a broad consensus among scientists regarding climate change?’ This required a Yes or No response in each experimental scenario.

Following this, participants completed the dependent variables using the same scales from the pilot study. These variables included the intention to perform pro‐environmental behaviours (*α* = .85), perceived behavioural control with a single item ‘Changing my behavior could have a positive and noticeable effect on climate change’, attitudes towards pro‐environmental behaviours (*α* = .71), willingness to participate in collective action against climate change (*α* = .89) and ideological orientation [*r*(671) = .67, *p* < .001]. As a manipulation check, participants were administered the same climate change scepticism scale used in the pilot study (*α* = .89). This scale includes items that challenge the consensus and scientific evidence on climate change.

Then, we proceeded to measure other scales that were not pertinent to the current investigation. Subsequently, we debriefed the participants and expressed our gratitude for their contribution.

### Results

#### Manipulation check

Participants in the denialist condition (*M* = 2.44, *SD* = 1.22) exhibited greater scepticism than those in the scientific consensus condition (*M* = 2.20, *SD* = 1.18), *t*(671) = −2.59, *p* = .014, 95% CI [0.04, 0.42], *d* = 0. 20. A subsequent regression analysis considering condition, gender and ideological orientation and their interactions as predictors revealed no significant interaction effects, *p* > .083 (see Supporting Information).

#### Regressions

We performed a series of regression analyses on the dependent variables using the module GAMLj in Jamovi. The experimental condition (0 for consensus, 1 for denialist) was the predictor. The moderators were gender (0 for women, 1 for men) and ideological orientation (centred). If significant interaction effects emerged, we conducted simple slope analyses using the two levels of gender (0 women, 1 men) and the 16th and 84th percentiles of the ideological orientation variable.

#### Intentions to perform pro‐environmental behaviours

**FIGURE 1:**
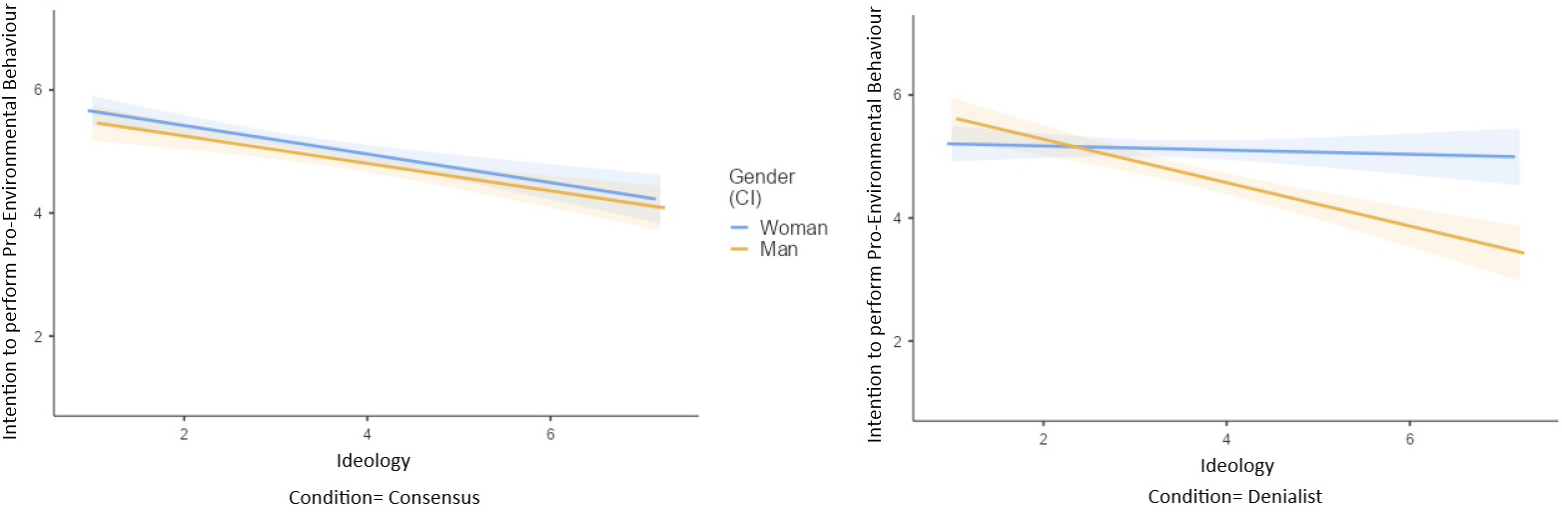
Interaction effect of experimental condition, gender, and ideological orientation on intention to perform pro‐environmental behaviours.

As presented in Table 3 and Figure 1, the three‐way interaction between condition, gender and ideological orientation was significant. The manipulation had an impact among left‐wing women and right‐wing men. Specifically, these groups showed fewer intentions to perform pro‐environmental behaviours in the denialist condition compared to the consensus condition. The manipulation did not affect left‐wing men nor right‐wing women. Also, a significant two‐way interaction effect between condition and ideological orientation emerged, although the conditional effects were not significant. The simple effects of gender and ideological orientation were also significant. Intentions to participate in pro‐environmental behaviours were weaker among men (*M* = 4.77, *SD* = 0.99) in comparison to women (*M* = 5.11, *SD* = 0.84) and as ideological orientation shifted towards the right.

**TABLE 3:** Effects of condition, gender, ideological orientation and their interactions on intention to perform pro‐environmental behaviours.

*R* ^2^ = .140						
Predictor	*b*.	*SE*	*p*	LLCI	ULCI	*β*
Constant	5.11	0.06	<.001	4.99	5.23	.00
Condition	0.02	0.09	.857	−0.16	0.20	.02
**Gender**	**−0.18**	**0.09**	.**044**	**−0.36**	**−0.01**	**−.19**
**Ideology**	**−0.23**	**0.05**	**<.001**	**−0.33**	**−0.14**	**−.32**
Condition × Gender	−0.18	0.14	.210	−0.45	0.10	−.19
**Condition** × **Ideology**	**0.20**	**0.07**	.**008**	**0.05**	**0.34**	.**27**
Left	−0.12	0.10	.253	−0.32	0.09	−.13
Right	−0.03	0.09	.706	−0.21	0.14	−.04
Gender × Ideology	0.01	0.07	.896	−0.13	0.14	.01
**Condition** × **Gender** × **Ideology**	**−0.33**	**0.11**	.**002**	**−0.54**	**−0.12**	**−.45**
**Woman‐left**	**−0.26**	**0.13**	.**045**	**−0.51**	**−0.01**	**−.28**
Woman‐right	0.23	0.13	.075	−0.02	0.49	.25
Man‐Left	0.02	0.16	.894	−0.30	0.34	.02
**Man‐Right**	**−0.30**	**0.12**	.**015**	**−0.55**	**−0.06**	**−.32**

*Note*: Significant effects appear in bold. Conditional effects appear indented.

Abbreviations: LLCI, lower‐level confidence interval; ULCI, upper‐level confidence interval.

##### Attitudes towards pro‐environmental behaviours

**FIGURE 2:**
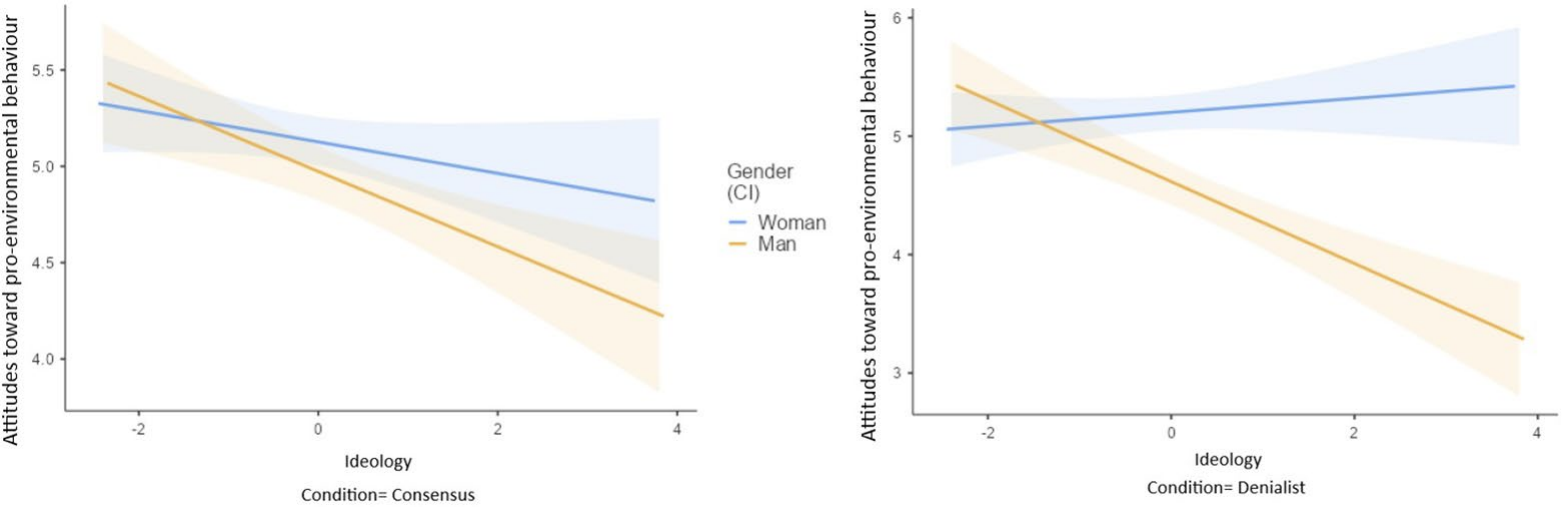
Interaction effect of experimental condition, gender and ideological orientation on attitudes towards pro‐environmental behaviours.

The three‐way interaction effect between condition, gender, and ideological orientation was significant (see Table 4 and Figure 2). The manipulation only influenced right‐wing men, who demonstrated less favourability towards pro‐environmental behaviours in the denialist condition compared to the consensus condition. The manipulation did not have an impact on the remainder groups (left‐wing and right‐wing women and left‐wing men). Furthermore, the interaction between condition and gender was significant, indicating that men (but not women) showed fewer positive attitudes in the denialist condition compared to the consensus condition.

**TABLE 4:** Effects of condition, gender, ideological orientation and their interactions on attitudes towards pro‐environmental behaviours.

*R* ^*2*^ = .117						
Predictor	*b*	SE	*p*	LLCI	ULCI	*β*
Constant	5.13	0.06	<.001	5.00	5.26	.00
Condition	0.07	0.10	.490	−0.13	0.26	.07
Gender	−0.17	0.10	.083	−0.36	0.02	−.17
**Ideology**	−0.08	0.05	.114	−0.18	0.02	−.11
**Condition** × **Gender**	**−0.43**	**0.15**	.**004**	**−0.73**	**−0.14**	**−.43**
Women	0.07	0.10	.490	−0.13	0.26	−.07
**Men**	**−0.36**	**0.12**	.**002**	**−0.59**	**0.14**	**−.37**
Condition × Ideology	0.14	0.08	.083	−0.02	0.30	.18
Gender × Ideology	−0.11	0.07	.126	−0.26	0.03	−.15
**Condition** × **Gender** × **Ideology**	**−0.29**	**0.12**	.**012**	**−0.52**	**−0.06**	**−.38**
Woman‐Left	−0.13	0.14	.360	−0.40	0.15	−.13
Woman‐Right	0.22	0.14	.116	−0.06	0.50	.22
Man‐Left	−0.15	0.18	.381	−0.50	0.19	−.15
**Man‐Right**	**−0.53**	**0.13**	**<.001**	**−0.79**	**−0.27**	**−.53**

*Note*: Significant effects appear in bold. Conditional effects appear indented.

Abbreviations: LLCI, lower‐level confidence interval; ULCI, upper‐level confidence interval.

##### Perceived behavioural control over pro‐environmental behaviours

**FIGURE 3:**
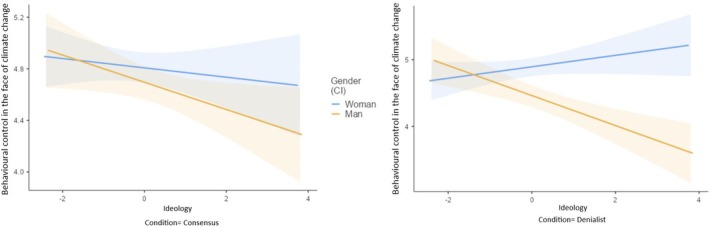
Interaction effect of condition, gender and ideological orientation on perceived behavioural control over pro‐environmental behaviours.

The effect of the three‐way interaction between condition, gender, and ideological orientation was significant (see Table 5 and Figure 3). Upon breaking down this interaction, it was observed that right‐wing men perceived lower behavioural control in the denialist condition than in the consensus condition. However, the manipulation did not have an impact on the remainder groups (left‐wing and right‐wing women and left‐wing men). The effect of the two‐way interaction between condition and gender was also significant, indicating that men (but not women) demonstrated lower perceived behavioural control in the denialist condition compared to the consensus condition.

**TABLE 5:** Effects of condition, gender, ideological orientation and their interactions on perceived behavioural control over pro‐environmental behaviours.

*R* ^*2*^ = .05						
Predictor	*b*	SE	*p*	LLCI	ULCI	*β*
Constant	4.81	0.06	<.001	4.69	4.93	.00
Condition	0.08	0.09	.380	−0.10	0.26	.09
Gender	−0.12	0.09	.185	−0.29	0.06	−.13
Ideology	−0.04	0.05	.451	−0.13	0.06	−.05
**Condition × Gender**	**−0.32**	**0.14**	.**024**	**−0.60**	**−0.04**	**−.35**
Women	0.08	0.09	.380	−0.10	0.26	.09
**Men**	**−0.24**	**0.11**	.**026**	**−0.45**	**−0.03**	**−.27**
Condition × Ideology	0.12	0.08	.104	−0.03	0.27	−.18
Gender × Ideology	−0.07	0.07	.315	−0.20	0.07	−.10
**Condition** × **Gender** × **Ideology**	**−0.24**	**0.11**	.**025**	**−0.45**	**−0.03**	**−.35**
Woman –Left	−0.09	0.13	.488	−0.34	0.16	−.10
Woman‐Right	0.22	0.13	.103	−0.04	0.47	.24
Man‐Left	−0.07	0.16	.661	−0.39	0.25	−.08
**Man‐Right**	**−0.37**	**0.13**	.**003**	**−0.62**	**−0.12**	**−.41**

*Note*: Significant effects appear in bold. Conditional effects appear indented.

Abbreviations: LLCI, lower‐level confidence interval; ULCI, upper‐level confidence interval.

##### Willingness to participate in collective action against climate change

**FIGURE 4:**
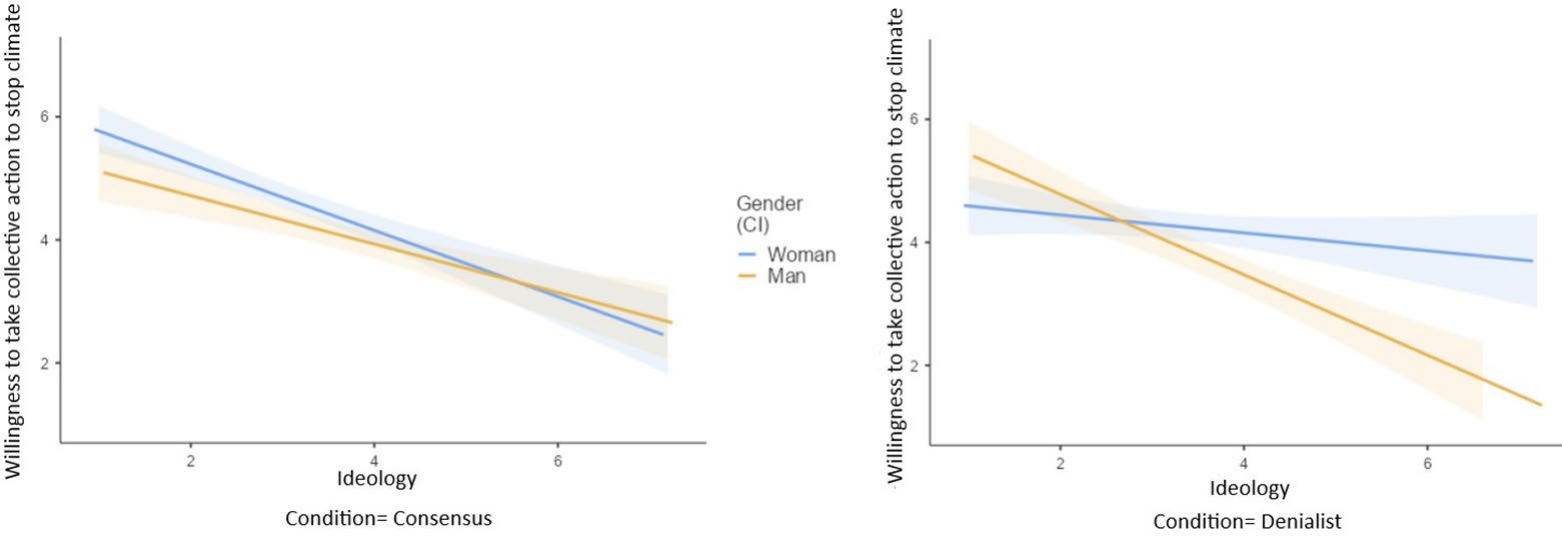
Interaction effect of condition, gender, ideological orientation on the willingness to participate in collective action against climate change.

The three‐way interaction between condition, gender and ideological orientation was significant (see Table 6 and Figure 4). The manipulation had an impact on left‐wing women and right‐wing men. In both groups, their willingness to participate in collective action was lower in the denialist condition than in the consensus condition. However, there were no differences between conditions for right‐wing women or left‐wing men. A significant two‐way interaction between condition and ideological orientation emerged, indicating that left‐wing (but not right‐wing) participants were less willing to participate in collective action in the denialist condition than in the consensus condition. The simple effects of gender and ideological orientation were also significant. The willingness to participate in collective action against climate change was weaker among men (*M* = 3.89, *SD* = 1.65) compared to women (*M* = 4.51, *SD* = 1.42) and as the ideological orientation moved towards the right.

**TABLE 6:** Effects of condition, gender, ideological orientation and their interactions on the willingness to participate in collective action against climate change.

*R* ^*2*^ = .18						
Predictor	*b*	SE	*p*	LLCI	ULCI	*β*
Constant	4.50	0.10	<.001	4.31	4.70	.00
Condition	−0.26	0.15	.089	−0.55	0.04	−.16
**Gender**	**−0.36**	**0.15**	.**015**	**−0.64**	**−0.07**	**−.23**
**Ideology**	**−0.54**	**0.08**	**<.001**	**−0.69**	**−0.38**	**−.44**
Condition × Gender	−0.06	0.23	.797	−0.51	0.39	−.04
**Condition** × **Ideology**	**0.39**	**0.12**	.**001**	**0.15**	**0.63**	.**32**
**Left**	**−0.38**	**0.17**	.**027**	**−0.71**	**−0.04**	**−.24**
Right	−0.21	0.15	.154	−0.50	0.08	−.13
Gender × Ideology	0.14	0.11	.199	−0.08	0.37	.12
**Condition** × **Gender** × **Ideology**	**−0.65**	**0.18**	**<.001**	**−1.00**	**−0.31**	**−.54**
**Woman‐Left**	**−0.80**	**0.21**	**<.001**	**−1.22**	**−0.39**	**−.51**
Woman‐Right	0.18	0.22	.409	−0.24	0.60	.11
Man‐Left	0.05	0.27	.854	−0.48	0.57	.03
**Man‐Right**	**−0.60**	**0.20**	.**003**	**−1.00**	**−0.20**	**−.38**

*Note*: Significant effects appear in bold. Conditional effects appear indented.

Abbreviations: LLCI, lower‐level confidence interval; ULCI, upper‐level confidence interval.

### Discussion

Study 1 suggested that gender and ideological orientation may moderate the effects of denying (vs. communicating) scientific consensus about human‐caused climate change. Specifically, right‐wing men exposed to denialist messages showed less intentions to engage in pro‐environmental behaviours, perceived less control over pro‐environmental behaviour, exhibited fewer positive attitudes towards pro‐environmental behaviour and were less willing to participate in collective action against climate change than right‐wing men exposed to scientific consensus. Unexpectedly, left‐wing women exposed to denialist messages also showed less intentions to engage in pro‐environmental behaviours and participate in collective action against climate change compared to those exposed to information about scientific consensus. However, since the effects of denialist messages on left‐wing women were inconsistent across the dependent variables—unlike the more consistent effects observed on right‐wing men—these results could simply reflect false positives.

A second experiment was conducted in Ecuador to verify the consistency and applicability of the effects observed in right‐wing men in a different cultural and socio‐economic context. This study also incorporated a control condition to ascertain whether attitudes and behavioural intentions were influenced more by messages denying consensus or those asserting its existence. Finally, the study was pre‐registered.

## STUDY 2: EFFECTS OF CONSENSUS‐DENYING AND CONSENSUS‐CONFIRMING MESSAGES: A REPLICATION AND EXTENSION IN ECUADOR

Study 2 aimed to validate the results from the previous study conducted in Spain within a non‐WEIRD context: Ecuador. Additionally, it sought to examine whether a denialist message leads to a negative impact or if a consensus message yields a positive effect compared to a control condition. This study utilized a similar methodology to Study 1 but introduced a control condition that did not impart any information about the presence or absence of scientific consensus on climate change.

We expected to find significant interaction effects between condition, gender and ideological orientation on the dependent variables. Right‐wing men were expected to have less intention to engage in pro‐environmental behaviours, demonstrate less favourability towards these behaviours, perceive less behavioural control to enact such behaviours and exhibit less willingness to participate in collective action against climate change in the denialist condition compared to the consensus and control conditions. In contrast, we did not anticipate significant differences between conditions among the other groups (left‐wing men and right‐wing and left‐wing women).

### Method

#### Participants

We recruited 365 Ecuadorian participants (60.5% women, average age; *M*_age_ = 28.93, *SD*_age_ = 9.48, age range = 17–60 years) via snowball technique and convenience sampling.

The survey was disseminated among the first author's contacts. Participation was voluntary and uncompensated. Although we initially received 387 complete responses, 22 were discarded for predefined reasons as stated in the pre‐registration: those that did not comply with the established time limit, either less than 200 s (*n* = 4) or more than 10,000 s (*n* = 18).

To estimate the statistical power to detect two specific three‐way interactions, we conducted post‐hoc power analyses using linear regression models in R. For each interaction, we specified a reduced model excluding the target three‐way interaction and a full model including it. The increase in explained variance (Δ*R* ^2^) attributed to the addition of the three‐way interaction was used to compute the effect size (*f* ^2^), which in turn was used in a power analysis via the pwr.f2.test function. Based on the sample size and observed effect sizes, these analysis revealed an estimated power of 0.681 to detect the three‐way interaction between Condition (denialist vs. consensus), Gender and Ideological Orientation, and an estimated power of 0.267 to detect the three‐way interaction between Condition (denialist vs. control), Gender and Ideological Orientation. These results indicate that the study had sufficient power to detect the first interaction but limited power to detect the second.

#### Procedure

Survey links were distributed, and participants were randomly allocated to one of three conditions: the scientific consensus condition, the message condition (which were identical to those in Study 1), or a control condition concerning a recent ash emission from an Ecuadorian volcano. As this was a replication of the prior study but with an enhanced control condition, the rest of the dependent variables were measured in the same way as before. These included scepticism towards climate change (*α* = .89), intention to perform pro‐environmental behaviours (*α* = .83), attitudes towards pro‐environmental behaviour (*α* = .83), perceived behavioural control over pro‐environmental actions (single item) and willingness to participate in collective action against climate change (*α* = .78). Ideological orientation was also measured as in previous studies [*r*(363) = .66, *p* < .001)].^1^

### Results

#### Manipulation check

Participants in the denialist condition exhibited greater scepticism (*M* = 2.96, *SD* = 1.37) compared to those in the control condition (*M* = 2.66, *SD* = 1.37), *t*(233) = −2.13, *p* = .043, 95% CI [−0.67, −0.02]. There were no significant differences in scepticism between the consensus condition (*M* = 2.73, *SD* = 1.33) and the denialist condition, *t*(247) = −1.34, *p* = .179, 95% CI [−0.55, −0.10], *d* = 0.22. A subsequent regression analysis considering condition, gender and ideological orientation and their interactions as predictors revealed no significant interaction effects, *p*s > .513 (see Supporting Information).

#### Regressions

To examine if gender and ideological orientation moderated the impact of the experimental manipulation on the dependent variables, we conducted moderation analyses using JAMOVI. As the experimental condition (the predictor) had three levels, we created two dummy variables: the first dummy (D1) compared the denialist message condition to the consensus condition (0 denialist, consensus 1), whereas the second dummy (D2) contrasted the denialist condition with the control one (0 denialist, control 1). The moderators included gender (0 women, 1 men) and ideological orientation (centred). If significant interactions were observed, we performed simple slope analyses with respect to the two levels of the gender variable (0 women, 1 men) and the 16th and 84th percentiles of the ideological orientation variable. Alternative analyses comparing the consensus condition and the control condition with each other are presented in Supporting Information.

##### Intention to perform pro‐environmental behaviours

**FIGURE 5:**
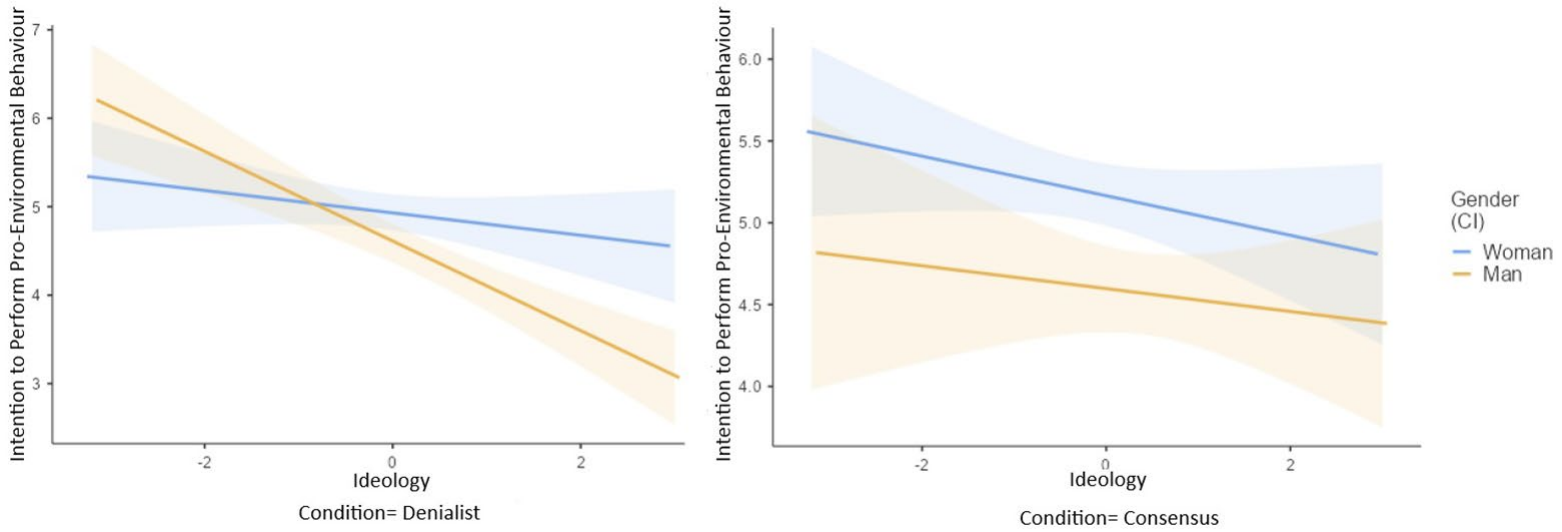
Interaction effect of experimental condition (denialist vs. consensus), gender and ideological orientation on intention to perform pro‐environmental behaviours.

As shown in Table 7, a significant three‐way interaction was observed between condition, gender and ideological orientation. This was significant in the comparison of the denialist condition with the consensus condition (D1) but not between the denialist and the control conditions (D2). Upon further examination, the manipulation only affected the behavioural intentions of right‐wing men who exhibited fewer intentions to perform pro‐environmental behaviours in the denialist condition than in the consensus one (see Figure 5). Moreover, a significant interaction effect between gender and ideological orientation was found. This interaction revealed that men showed fewer pro‐environmental behavioural intentions than women, but these differences were greater for right‐wing individuals than for left‐wing individuals. Additionally, the simple effect of gender was also significant. Men showed a lower intention to perform pro‐environmental behaviours (*M* = 4.55, *SD* = 0.91) than women (*M* = 4.96, *SD* = 0.88).

**TABLE 7:** Effects of condition, gender, ideological orientation and their interactions on the intention to perform pro‐environmental behaviours.

*R* ^2^ = .173						
Predictor	*b*	SE	*p*	LLCI	ULCI	*β*
Constant	4.94	0.10	<.001	4.73	5.14	<.001
D1	0.23	0.14	.096	−0.04	0.51	.26
D2	−0.12	0.14	.408	−0.40	0.16	−.13
**Gender**	**−0.35**	**0.15**	.**021**	**−0.65**	**−0.05**	**−.38**
Ideology	−0.13	0.10	.200	−0.32	0.07	−.16
D1 × Gender	−0.23	0.22	.309	−0.67	0.21	−.25
D2 × Gender	0.30	0.23	.194	−0.15	0.74	.32
D1 × Ideology	0.01	0.13	.964	−0.25	0.26	.01
D2 × Ideology	0.13	0.14	.354	−0.14	0.39	.16
**Gender** × **Ideology**	**−0.38**	**0.13**	.**004**	**−0.64**	**−0.12**	**−.48**
**Left**	**−0.22**	**0.11**	.**043**	**−0.44**	**−0.01**	**−.24**
**Right**	**−0.46**	**0.11**	**<.001**	**−0.68**	**−0.23**	**−.50**
**D1** × **Gender** × **Ideology**	**0.43**	**0.19**	.**026**	**0.05**	**0.81**	.**54**
Woman‐Left	0.23	0.15	.132	−0.07	0.53	.25
Woman‐Right	0.24	0.19	.206	−0.13	0.61	.26
Man‐Left	−0.29	0.22	.192	−0.72	0.14	−.31
**Man‐Right**	**0.37**	**0.18**	.**044**	**0.01**	**0.73**	.**40**
D2 × Gender × Ideology	0.24	0.20	.222	−0.15	0.64	.31
Woman‐Left	−0.20	0.17	.224	−0.53	0.13	−.22
Woman‐Right	−0.01	0.19	.937	−0.38	0.35	−.02
Man‐Left	−0.07	0.20	.719	−0.46	0.32	−.08
**Man‐Right**	**0.48**	**0.22**	.**028**	**0.05**	**0.91**	.**53**

*Note*: Significant effects appear in bold. Conditional effects appear indented.

Abbreviations: LLCI, lower‐level confidence interval; ULCI, upper‐level confidence interval.

##### Attitudes towards pro‐environmental behaviours

The effect of the three‐way interaction between condition, gender and ideological orientation was significant (see Table 8) in comparison between the denialist condition and the consensus condition (D1). However, no conditional effects were significant.

**TABLE 8:** Effects of condition, gender, ideological orientation and their interactions in attitudes towards pro‐environmental behaviours.

*R* ^2^ = .09						
Predictor	*b*	SE	*p*	LLCI	ULCI	*β*
Constant	4.76	0.14	<.001	4.48	5.04	<.001
D1	0.05	0.20	.790	−0.33	0.44	.04
D2	0.03	0.20	.878	−0.36	0.42	.03
Gender	−0.31	0.21	.137	−0.73	0.10	−.26
Ideology	−0.17	0.14	.207	−0.44	0.10	−.16
D1 × Gender	−0.10	0.31	.741	−0.71	0.51	−.08
D2 × Gender	0.39	0.32	.221	−0.23	1.01	.32
D1 × Ideology	0.01	0.18	.956	−0.34	0.36	.01
D2 v Ideology	0.27	0.19	.148	−0.10	0.64	.26
Gender × Ideology	−0.35	0.18	.057	−0.71	0.01	−.33
**D1** × **Gender** × **Ideology**	**0.56**	**0.27**	.**039**	**0.03**	**1.08**	.**52**
Woman‐Left	0.05	0.21	.831	−0.37	0.44	.04
Woman‐Right	0.06	0.26	.819	−0.46	0.58	.05
Man‐Left	−0.43	0.31	.159	−1.03	0.17	−.36
Man‐Right	0.42	0.25	.101	−0.08	0.92	.34
D2 × Gender × Ideology	0.11	0.28	.685	−0.43	0.66	.11
Woman‐Left	−0.15	0.23	.510	−0.61	0.30	−.13
Woman‐Right	0.26	0.26	.320	−0.25	0.76	.21
Man‐Left	0.16	0.28	.565	−0.38	0.70	.13
**Man‐Right**	**0.74**	**0.30**	.**016**	**0.14**	**1.34**	.**61**

*Note*: Significant effects appear in bold. Conditional effects appear indented.

Abbreviations: LLCI, lower‐level confidence interval; ULCI, upper‐level confidence interval.

##### Perceived behavioural control over pro‐environmental behaviours

No significant main or interaction effects emerged (see Table 9).

**TABLE 9:** Effects of condition, gender, ideological orientation and their interactions on perceived behavioural control over pro‐environmental behaviours.

*R* ^2^ = .068						
Predictor	*b*	*SE*	*p*	LLCI	ULCI	*β*
Constant	4.49	0.19	<.001	4.12	4.86	−.05
D1	0.01	0.26	.954	−0.49	0.52	.01
D2	0.37	0.26	.158	−0.14	0.89	.24
Gender	−0.31	0.28	.264	−0.85	0.23	−20
Ideology	−0.11	0.18	.546	−0.46	0.25	−.08
D1 × Gender	0.30	0.41	.462	−0.50	1.10	.19
D2 × Gender	0.52	0.42	.212	−0.30	1.34	.33
D1 × Ideology	0.20	0.24	.403	−0.27	0.66	.14
D2 × Ideology	0.28	0.25	.267	−0.21	0.76	.20
Gender × Ideology	−0.28	0.24	.246	−0.76	0.19	−.21
D1 × Gender × Ideology	0.29	0.35	.417	−0.41	0.98	.21
D2 × Gender × Ideology	0.10	0.36	.789	−0.62	0.82	.07

Abbreviations: LLCI, lower‐level confidence interval; ULCI, upper‐level confidence interval.

##### Willingness to participate in collective action against climate change

There only was a significant effect of gender, indicating that women exhibited a stronger willingness to participate in collective action (*M* = 4.37, *SD* = 1.30) than men (*M* = 3.87, *SD* = 1.34) (see Table 10).

**TABLE 10:** Effects of condition, gender, ideological orientation and their interactions on willingness to participate in collective action against climate change.

*R* ^2^ = .111						
Predictor	*b*	*SE*	*p*	LLCI	ULCI	*β*
Constant	4.50	0.16	<.001	4.19	4.81	<.001
D1	−0.17	0.22	.435	−0.59	0.25	−.13
D2	−0.30	0.22	.172	−0.73	0.13	−.22
**Gender**	**−0.52**	**0.23**	.**024**	**−0.98**	**−0.07**	**−.39**
Ideology	−0.25	0.15	.096	−0.55	0.04	−.22
D1 × Gender	0.18	0.34	.600	−0.49	0.85	.13
D2 × Gender	0.23	0.35	.506	−0.46	0.92	.17
D1 × Ideology	0.07	0.20	.722	−0.32	0.46	.06
D2 × Ideology	0.07	0.21	.723	−0.33	0.48	.06
Gender × Ideology	−0.28	0.20	.175	−0.67	0.12	−.24
D1 × Gender × Ideology	0.27	0.30	.369	−0.32	0.85	.23
D2 × Gender × Ideology	0.28	0.31	.355	−0.32	0.89	.24

*Note*: Significant effects appear in bold.

Abbreviations: LLCI, lower‐level confidence interval; ULCI, upper‐level confidence interval.

### Discussion

Consistently with Study 2, right‐wing men exposed to a denial message disputing scientific consensus exhibited less intention to perform pro‐environmental behaviours compared to those who received messages confirming scientific consensus regarding the anthropocentric origin of climate change. No other demographic group (including left‐wing or right‐wing women and left‐wing men) altered their behavioural intentions following the manipulation. However, the results of Study 1 were not echoed for this group on attitudes, perceived control, or collective action intentions. Additionally, we found no significant differences between the denialist and control conditions, nor between the consensus and control conditions (as shown in the Supporting Information).

One possible explanation for the differences between the results of Studies 1 and 2 is that the latter lacked the statistical power to detect three‐way interaction effects. Supporting this idea, a breakdown of some non‐significant interactions showed significant conditional effects consistent with the expected effects among right‐wing men in the comparison between the denialist and control conditions (e.g. behavioural intentions and attitudes). Another possibility is that the manipulation was less impactful in Ecuador than in Spain, potentially due to cultural or socio‐economic differences. Future cross‐cultural research could help clarify the role of country‐level variables in shaping these effects.

## GENERAL DISCUSSION

This research investigated the impact of messages denying the scientific consensus on anthropogenic climate change on pro‐environmental intentions and other correlates, focusing on the moderating roles of gender and ideological orientation. Across two experimental studies conducted in Spain and Ecuador, we found consistent evidence that a single exposure to denialist messages had a detrimental effect on right‐wing men's intentions to perform pro‐environmental behaviours. In Studies 1 and 2, right‐wing men exposed to a denialist message showed weaker intentions to perform pro‐environmental behaviour than those who received consensus‐affirming messages. Study 1 also found negative impacts on right‐wing men's perceived control and attitudes towards these behaviours, as well as on willingness to participate in collective action to mitigate climate change, though these effects were not replicated in Study 2. In contrast, we did not find consistent effects of climate misinformation on women or left‐wing men across studies.

The absence of consistent effects among women and left‐wing men, alongside the significant impact of denialist messages on right‐wing men across our studies, further reinforces the idea that ideological alignment and gendered worldviews may shape how individuals interpret and respond to climate‐related information (see Gozzer & Domínguez, 2011; McCright, 2010; McCright & Dunlap, 2011; Xiao & McCright, 2012). The effects of denialist messages on right‐wing men could be motivated by the desire to protect the status quo and resistance to change, especially if proposed solutions are perceived as threatening to personal values or interests (Feygina et al., 2010). The vulnerability of right‐wing men to denialist information might also be attributed to the activation of the fluency heuristic, which suggests that messages frequently circulated in right‐wing media (Björnberg et al., 2017) would be more easily processed, leading to increased perceived plausibility. This, in turn, could reduce pro‐environmental behavioural intentions (Imundo & Rapp, 2022; McCright, 2016; Spampatti et al., 2024; Van der Linden et al., 2015). Future studies may explore these explanations through empirical testing.

Our findings reveal some parallels and distinctions when compared to previous research. Consistent with Cook et al. (2017), who found that free‐market ideology moderated responses to consensus (mis)information, our results also highlight the crucial role of ideological orientation. However, our study extends this by identifying a specific vulnerability among right‐wing men, an interaction involving gender that was not reported as a key moderator by Cook et al. Additionally, a key distinction lies in the outcome variables: while much previous research predominantly examined attitudinal changes, our study measured both attitudes *and* behavioural intentions. This methodological difference is relevant as our findings indicated that while effects on attitudes were less consistent across different conditions, the impacts on behavioural intentions demonstrated greater consistency. Furthermore, while most of the experimental research on this topic was developed within WEIRD contexts, we include a non‐WEIRD population (Ecuadorian). Observing ideological moderation across these different contexts and specific populations reinforces the hypothesis that worldview may significantly shape responses to climate communication.

It is noteworthy that the differences in behavioural intentions between conditions among right‐wing men did not appear to be strictly contingent upon a preceding shift in climate scepticism. The interaction effect between condition, gender, and ideological orientation that was consistently significant for behavioural intentions across studies did not emerge for scepticism. This finding suggests that exposure to denialist messages might influence the willingness to act pro‐environmentally through pathways that do not necessarily involve a significant, measurable change in overt scepticism first. For example, such messages might serve more as a justification or a convenient cue to disengage from effortful pro‐environmental actions for those already predisposed against them or subtly alter perceived social norms within their reference group, thereby impacting intentions.

The discovery that messages denying (vs. affirming) scientific consensus adversely impact the intentions of right‐wing men to engage in climate change mitigation behaviours is concerning. It is not merely that a large portion of the population, namely right‐wing men, refrain from individual actions conducive to combating climate change due to exposure to denialist messages. More critically, this group seems to have considerable sway in the political arena, potentially hampering prompt systemic decision‐making essential for effectively mitigating the climate emergency. Men are generally more politically involved than women—they constitute 77.2% of cabinet members who manage ministries and policy spheres, are notably overrepresented in the realm of political lobbying (Junk et al., 2021), and particularly in business lobbies where 88% are men (Vitón García, 2017). Combating climate emergencies calls for not just individual interventions but also structural changes (Calvin et al., 2023). However, the inclination of right‐wing men towards denialist messages could delay these measures. Strategies to counter the effects of disinformation such as prebunking and debunking (Lewandowsky & van der Linden, 2021; Schubatzky & Haagen‐Schützenhöfer, 2022) should be tailored to right‐wing men, who appear to be most vulnerable to this type of misinformation.

We wish to highlight a few limitations in our studies that might be addressed in future research. Although our research enhances our understanding of the impact of denialist messages in lesser‐studied contexts, some effects observed in the Spanish sample (Study 1) were not fully replicated in the Ecuadorian sample (Study 2). As aforementioned, the manipulation could have had a lesser impact on the Ecuadorian sample due to cultural or socio‐economic differences.

Secondly, a key limitation involves the comparisons with the control group in Study 2. While we observed differences in the behavioural intentions of right‐wing men between the denialist and consensus‐affirming conditions, no significant differences emerged when comparing the denialist condition to the neutral control condition. Thus, our most robust finding concerns the relative impact of denial versus consensus messages, rather than demonstrating a clear shift from a neutral baseline. We suspect this lack of significance against the control may partly reflect insufficient statistical power for detecting this complex interaction with our sample size in Study 2. Tentatively supporting this, some specific conditional effects within the non‐significant overall interaction (e.g. the comparison between denial and control for right‐wing men's intentions and attitudes) did align with our hypotheses. Further studies with larger sample sizes may shed light on significant effects we failed to detect.

We also acknowledge that using convenience and snowball sampling may limit the generalizability of our results. For instance, given the polarization of climate change beliefs along political lines, the absence of a fully representative sample may have skewed our results. Specifically, the observed reduction in pro‐environmental behavioural intentions among right‐wing men may manifest differently in a more politically balanced population. Future studies should strive for more politically representative samples. Another limitation is that we measured behavioural intentions rather than actual behaviour. Future work should consider introducing a behavioural measurement to investigate if denialist messages indeed undermine environmental behaviours. Furthermore, longitudinal studies are needed to examine whether exposure to climate misinformation has lasting effects on pro‐environmental intentions and behaviours. Finally, future research could also explore the psychological mechanisms underlying the interaction between gender, ideological orientation and susceptibility to climate misinformation.

## CONCLUSION

In conclusion, this research provides causal evidence that messages denying the scientific consensus on anthropogenic climate change can significantly reduce pro‐environmental intentions. Crucially, our findings also underscore the moderating role of gender and ideological orientation in shaping individuals' susceptibility to climate misinformation. Across two culturally distinct contexts, Spain and Ecuador, we consistently found that denialist (versus consensus‐affirming) messages diminished pro‐environmental behavioural intentions among right‐wing men, while effects on women and left‐wing men were inconsistent or absent. These results point to a specific vulnerability among right‐wing men, a group with disproportionate influence in political and economic domains. Notably, the observed effects on behavioural intentions appeared to occur independently of measurable changes in climate scepticism, emphasizing the need for further research into the psychological mechanisms through which misinformation exerts its influence. By extending previous research to include behavioural intentions and incorporating data from a non‐WEIRD population, this study contributes novel insights into the complex interplay between ideology, gender, and climate misinformation.

## AUTHOR CONTRIBUTIONS

**Aitor Larzabal‐Fernandez:** Conceptualization; investigation; funding acquisition; writing – original draft; methodology; validation; visualization; writing – review and editing; software; formal analysis; project administration; data curation; supervision; resources. **Alexandra Vázquez:** Data curation; supervision; resources; software; formal analysis; project administration; methodology; validation; visualization; writing – review and editing; writing – original draft; funding acquisition; investigation; conceptualization. **Angela Castrechini Trotta:** Funding acquisition; visualization; validation.

## CONFLICT OF INTEREST STATEMENT

The authors declare that there are no conflicts of interest related to this study.

## Supporting information


Data S1.

## Data Availability

The preregistration of the study, including detailed methods and analysis plans, is available upon the Open Science Framework at https://osf.io/th7pc?view_only=4b3bd36be32940da8a5c3a01bbe9766f. Data are available from the corresponding author upon reasonable request.

## Footnotes

^1^
In the pre‐registration, we also measured the Right‐Wing Authoritarianism (RWA) as a potential moderator, similarly to ideological orientation. However, since the scale was found to be unreliable, the analyses were discarded.
