# Supplementary material for: Gender and ideological orientation moderate the influence of climate misinformation on pro‐environmental behavioural intentions
Source: Br J Soc Psychol. 2025 Jul 1;64(3):e70000. doi: 10.1111/bjso.70000 (PMC12210336; doi:10.1111/bjso.70000)
Supplement: Supplementary file 1 — Data S1. [file BJSO-64-0-s001.docx]

**SUPPLEMENTARY MATERIALS**

[**1. Factor analysis: Intention to Perform Pro-Environmental Behaviors 2**](#_Toc198196367)

[**2. Correlation analyses subdivided by Gender 3**](#_Toc198196368)

[**3. Manipulation check Study 1 Spain 4**](#_Toc198196369)

[**4. Separate analysis for subcategories of the instrument “Intention to Perform Pro-Environmental Behaviors” in Spain 5**](#_Toc198196370)

[**5. Manipulation check Study 2 Ecuador 8**](#_Toc198196371)

[**6. Separate analysis for subcategories of the instrument “Intention to Perform Pro-Environmental Behaviors” in Ecuador 10**](#_Toc198196372)

[**7. Study 2 Ecuador: Alternative analyses comparing the consensus condition and the control condition 12**](#_Toc198196373)

# Factor analysis: Intention to Perform Pro-Environmental Behaviors

In the preliminary exploratory factor analysis, factorial adequacy tests showed adequate Kaiser-Meyer-Olkin (KMO) values ​​(KMO = 853 and Bartlett’s sphericity test (*X* ^2^_(105)_ = 3753.687; *p* < .001), which indicates good adaptation. In the analysis, three factors were found that explained 61.64% of the variance.

**Table 1.**

*Component Matrix*

| **No.** | **Item** | **Factor** | | |
| --- | --- | --- | --- | --- |
|  |  | **Reduce and recycle** | **Diet** | **Transport** |
| **1** | Living without a car |  |  | .801 |
| **2** | Use electric car |  |  | .405 |
| **3** | Have a vegan diet. |  | .881 |  |
| **4** | To use the car less |  |  | .728 |
| **5** | Have a vegetarian diet. |  | .899 |  |
| **6** | Eat organic food |  | .573 |  |
| **7** | Buy smaller cars |  |  | .552 |
| **8** | Reduce food waste | .869 |  |  |
| **9** | Reduce food waste | .846 |  |  |
| **10** | Buy food without unnecessary packaging | .759 |  |  |
| **11** | Eat less meat |  | .695 |  |
| **12** | Buy less clothes | .425 |  |  |
| **13** | Use recycled materials | .781 |  |  |
| **14** | Use less paper | .710 |  |  |
| **15** | Recycle | .725 |  |  |

The scale had three dimensions: behaviors related to transportation (“Living without a car”, α = 0.73 and ω = 0.75), diet-related behaviors (“Eat organic food”, α = 0.81 and ω = 0.83) and behaviors related to reducing consumption and recycling (“Buy fewer clothes”, α = 0.84).

# Correlation Analyses Subdivided by Gender

Comparison of correlations

To check whether the strength of the associations between the variables of interest is different for men and women, we carried out a correlation analysis for each group (Table 2).

**Table 2.**

*Comparison of Correlations*

|  | 1 | 2 | 3 | 4 | 5 | 6 | 7 | 8 |
| --- | --- | --- | --- | --- | --- | --- | --- | --- |
| 1 Climate Skepticism | 1 | -.37^**^ | -.27^**^ | -.27^**^ | -.42^**^ | -.42^**^ | -.14^*^ | -.32^**^ |
| 2 Intention to Perform Pro-Environmental Behaviors | -.52 ^**^ | 1 | .80^**^ | .79^**^ | .80^**^ | .55^**^ | .11 | .53^**^ |
| 3 Transport intention | -.51^**^ | .79^**^ | 1 | .43^**^ | .57^**^ | .43^**^ | .14^**^ | .44^**^ |
| 4 Diet Intention | -.36^**^ | .77^**^ | .51 ^**^ | 1 | .43^**^ | .39^**^ | .09 | .47^**^ |
| 5 Intention to recycle and reduce | -.44^**^ | .84^**^ | .49 ^**^ | .45^**^ | 1 | .54^**^ | .04 | .38^**^ |
| 6 Attitudes Towards Pro-Environmental Behaviors | -.41^**^ | .58^**^ | .40^**^ | .45^**^ | .55^**^ | 1 | .11 | .48^**^ |
| 7 Perceived Behavioral Control over Pro-Environmental Behaviors. | -.22^**^ | .33^**^ | .24^**^ | .21^**^ | .32 ^**^ | .41^**^ | 1 | .13 |
| 8 Willingness to Participate in Collective Action Against Climate Change. | -.54^**^ | .66^**^ | .55^**^ | .59^**^ | .52 ^**^ | .56^**^ | .22^**^ | 1 |

*Note*. Correlations for women are shown above the diagonal, and correlations for men are shown below the diagonal. *The correlation is significant at the .05 level; **. The correlation is significant at the < .01 level; *n =* 406

# Manipulation Check Study 1 Spain

| 1. Model Fit | | | | | |
| --- | --- | --- | --- | --- | --- |
| **R²** | **Adj. R²** | **df** | **df (res)** | **F** | **p** |
| 0.17 | 0.17 | 7 | 665 | 19.99 | <.001 |

| Parameter Estimates (Coefficients) | | | | | | | | | |
| --- | --- | --- | --- | --- | --- | --- | --- | --- | --- |
|  | | | | **95% Confidence Intervals** | |  | | | |
| **Names** | **Effect** | **Estimate** | **SE** | **Lower** | **Upper** | **β** | **df** | **t** | **p** |
| (Intercept) | (Intercept) | 2.32 | 0.04 | 2.23 | 2.41 | 0.02 | 665 | 51.85 | <.001 |
| Condition | Skepticism - No skepticism | 0.26 | 0.09 | 0.08 | 0.43 | 0.21 | 665 | 2.87 | 0.004 |
| Gender | Man - Woman | 0.19 | 0.09 | 0.01 | 0.36 | 0.15 | 665 | 2.07 | 0.039 |
| Ideology | Ideology | 0.36 | 0.03 | 0.29 | 0.43 | 0.38 | 665 | 10.53 | <.001 |
| Condition ✻ Gender | (Skepticism - No skepticism) ✻ (Man - Woman) | 0.21 | 0.18 | -0.14 | 0.56 | 0.17 | 665 | 1.18 | 0.236 |
| Condition ✻ Ideology | (Skepticism - No skepticism) ✻ Ideology | -0.01 | 0.07 | -0.14 | 0.13 | -0.01 | 665 | -0.13 | 0.897 |
| Gender1✻ Ideology | (Man - Woman) ✻ Ideology | 0.12 | 0.07 | -0.02 | 0.25 | 0.13 | 665 | 1.74 | 0.083 |
| Condition ✻ Gender1✻ Ideology | (Skepticism - No skepticism) ✻ (Man - Woman) ✻ Ideology | 0.09 | 0.14 | -0.18 | 0.36 | 0.10 | 665 | 0.65 | 0.513 |

| Parameter Estimates for simple effects of Condition | | | | | | | | | | |
| --- | --- | --- | --- | --- | --- | --- | --- | --- | --- | --- |
| **Moderator** | |  | | | **95% Confidence Intervals** | |  | | | |
| **Ideology** | **Gender** | **Effect** | **Estimate** | **SE** | **Lower** | **Upper** | **β** | **df** | **t** | **p** |
| **50-34%** | **Woman** | Denialist - Consensus | 0.23 | 0.16 | -0.10 | 0.55 | 0.19 | 665 | 1.37 | 0.171 |
|  | **Man** | Denialist - Consensus | 0.31 | 0.21 | -0.10 | 0.72 | 0.26 | 665 | 1.50 | 0.133 |
| **50%** | **Woman** | Denialist - Consensus | 0.15 | 0.12 | -0.09 | 0.38 | 0.12 | 665 | 1.23 | 0.220 |
|  | **Man** | Denialist - Consensus | 0.37 | 0.13 | 0.10 | 0.63 | 0.30 | 665 | 2.73 | 0.007 |
| **50+34%** | **Woman** | Denialist - Consensus | 0.09 | 0.17 | -0.24 | 0.42 | 0.08 | 665 | 0.55 | 0.583 |
|  | **Man** | Denialist - Consensus | 0.40 | 0.16 | 0.09 | 0.71 | 0.33 | 665 | 2.54 | 0.011 |

# Separate Analysis for Subcategories of the Instrument “Intention to Perform Pro-Environmental Behaviors” in Spain (Study 1)

*Effects of Condition, Gender, Ideological Orientation and Their Interactions on Intention to Recycle and Reduce Consumption*

| **Predictor** | ***b*** | ***HE*** | ***β*** | ***t*** | ***p*** | **LLCI** | **ULCI** |
| --- | --- | --- | --- | --- | --- | --- | --- |
| **Constant** | **6.17** | **0.06** | **-0.01** | **99.96** | **<.001** | **6.05** | **6.29** |
| Condition | 0.11 | 0.09 | -0.01 | 1.22 | .222 | -0.07 | 0.30 |
| Gender | -0.08 | 0.09 | -0.22 | -0.89 | .370 | -0.26 | 0.09 |
| **Ideology** | **-0.12** | **0.04** | **-0.20** | **-2.58** | **.010** | **-0.22** | **-0.03** |
| Condition X Gender | -0.24 | 0.14 | -0.26 | -1.67 | .094 | -0.52 | 0.04 |
| Condition X Ideology | 0.11 | 0.07 | 0.06 | 1.43 | .150 | -0.04 | 0.26 |
| Gender X Ideology | 0.00 | 0.07 | -0.20 | 0.06 | .946 | -0.13 | 0.14 |
| **Condition X Gender X Ideology** | **-0.30** | **0.11** | **-0.42** | **-2.74** | **.006** | **-0.52** | **-0.09** |
| Woman -Left | -0.04 | 0.13 | -0.04 | -0.29 | .764 | -0.30 | 0.22 |
| Woman-Right | 0.23 | 0.13 | 0.26 | 1.75 | .079 | -0.02 | 0.50 |
| Man-Left | 0.14 | 0.16 | 0.15 | 0.84 | .400 | -0.18 | 0.47 |
| **Man-Right** | **-0.34** | **0.12** | **-0.36** | **-2.64** | **.008** | **-0.59** | **-0.08** |

*Notes*. LLCI = Lower level confidence interval; ULCI = upper-level confidence interval. Significant effects appear in bold. Conditional effects appear indented.

*Effects of Condition, Gender, Ideological Orientation and Their Interactions on Intention to Modify Transportation Habits*

| **Predictor** | ***b*** | ***HE*** | ***β*** | ***t*** | ***p*** | **LLCI** | **ULCI** |
| --- | --- | --- | --- | --- | --- | --- | --- |
| **Constant** | **4.99** | **0.08** | **-0.09** | **59.09** | **<.001** | **4.82** | **5.15** |
| Condition | 0.00 | 0.12 | 0.00 | 0.02 | .983 | -0.25 | 0.26 |
| Gender | -0.11 | 0.12 | -0.09 | -0.87 | .382 | -0.35 | 0.14 |
| **Ideology** | **-0.18** | **0.06** | **-0.19** | **-2.78** | **.005** | **-0.32** | **-0.05** |
| Condition X Gender | -0.25 | 0.19 | -0.20 | -1.30 | .193 | -0.64 | 0.13 |
| Condition X Ideology | 0.18 | 0.10 | 0.01 | 1.74 | .081 | -0.02 | 0.39 |
| **Gender X Ideology** | -0.08 | 0.09 | -0.09 | -0.87 | .383 | -0.27 | 0.11 |
| **Condition X Gender X Ideology** | **-0.35** | **0.15** | **-0.35** | **-2.31** | **.020** | **-0.64** | **-0.05** |
| Woman -Left | -0.25 | 0.18 | -0.20 | -1.39 | .163 | -0.61 | 0.10 |
| Woman-Right | 0.20 | 0.18 | 0.16 | 1.10 | .267 | -0.15 | 0.57 |
| Man-Left | -0.02 | 0.23 | -0.02 | -0.09 | .922 | -0.47 | 0.43 |
| **Man-Right** | **-0.43** | **0.17** | **-0.34** | **-2.49** | **.012** | **-0.78** | **-0.09** |

*Notes*. LLCI = Lower level confidence interval; ULCI = upper-level confidence interval. Significant effects appear in bold. Conditional effects appear indented.

*Effects of Condition, Gender, Ideological Orientation and Their Interactions on*

*Intention to Modify Diet*

| **Predictor** | ***b*** | ***HE*** | ***β*** | ***t*** | ***p*** | **LLCI** | **ULCI** |
| --- | --- | --- | --- | --- | --- | --- | --- |
| **Constant** | **4.21** | **0.10** | **0.17** | **41.84** | **<.001** | **4.01** | **4.41** |
| Condition | -0.07 | 0.15 | -0.05 | -0.51 | .605 | -0.38 | 0.22 |
| **Gender** | **-0.45** | **0.15** | **-0.29** | **-3.03** | **.002** | **-0.75** | **-0.16** |
| **Ideology** | **-0.47** | **0.08** | **-0.39** | **-5.88** | **<.001** | **-0.63** | **-0.31** |
| Condition X Gender | -0.18 | 0.23 | -0.12 | -0.79 | .424 | -0.65 | 0.27 |
| **Condition X Ideology** | **0.33** | **0.12** | **0.28** | **2.67** | **.007** | **0.08** | **0.58** |
| **Left** | **-0.55** | **0.21** | **-0.25** | **-2.53** | **.011** | **-0.98** | **-0.12** |
| Right | 029 | 0.22 | 0.00 | 1.31 | .187 | -0.14 | 0.72 |
| Gender X Ideology | 0.07 | 0.11 | 0.06 | 0.67 | .502 | -0.14 | 0.30 |
| Condition X Gender X Ideology | -0.34 | 0.18 | -0.28 | -1.91 | .056 | -0.70 | 0.00 |
| **Woman -Left** | **-0.55** | **0.21** | **-0.35** | **-2.53** | **.011** | **-0.98** | **-0.12** |
| Woman-Right | 0.29 | 0.22 | 0.18 | 1.31 | .187 | -0.14 | 0.72 |
| Man-Left | -0.25 | 0.27 | -0.16 | -0.93 | .350 | -0.79 | 0.28 |
| Man-Right | -0.27 | 0.21 | -0.18 | -1.32 | .185 | -0.69 | 0.13 |

*Notes*. LLCI = Lower level confidence interval; ULCI = upper-level confidence interval. Significant effects appear in bold. Conditional effects appear indented.

# Manipulation Check Study 2 Ecuador

| Model Fit | | | | | |
| --- | --- | --- | --- | --- | --- |
| **R²** | **Adj. R²** | **df** | **df (res)** | **F** | **p** |
| 0.15 | 0.12 | 11 | 345 | 5.60 | <.001 |

| Parameter Estimates (Coefficients) | | | | | | | | | |
| --- | --- | --- | --- | --- | --- | --- | --- | --- | --- |
|  | | | | **95% Confidence Intervals** | |  | | | |
| **Names** | **Effect** | **Estimate** | **SE** | **Lower** | **Upper** | **β** | **df** | **t** | **p** |
| (Intercept) | (Intercept) | 2.61 | 0.15 | 2.32 | 2.90 | -0.13 | 345 | 17.49 | <.001 |
| Gender | Men - Women | 0.59 | 0.22 | 0.16 | 1.03 | 0.45 | 345 | 2.67 | 0.008 |
| D1 | Consensus - Denialist | -0.14 | 0.21 | -0.54 | 0.26 | -0.11 | 345 | -0.68 | 0.495 |
| D2 | Control - Dnialist | -0.10 | 0.21 | -0.51 | 0.32 | -0.07 | 345 | -0.46 | 0.648 |
| Ideology | Ideology | 0.30 | 0.14 | 0.02 | 0.58 | 0.26 | 345 | 2.07 | 0.039 |
| Gender ✻ D1 | (Men -Women) ✻ (Consensus - Denialist) | 0.08 | 0.33 | -0.57 | 0.72 | 0.06 | 345 | 0.23 | 0.820 |
| Gender ✻ D2 | (Men - Women) ✻ (Control - Denialist) | -0.09 | 0.34 | -0.75 | 0.57 | -0.07 | 345 | -0.26 | 0.794 |
| D1 ✻ Ideology | (Consensus - Denialist) ✻ Ideology | -0.25 | 0.19 | -0.61 | 0.12 | -0.22 | 345 | -1.32 | 0.188 |
| D2 ✻ Ideology | (Control - Denialist) ✻ Ideology | -0.11 | 0.20 | -0.50 | 0.28 | -0.10 | 345 | -0.56 | 0.575 |
| Gender ✻ Ideology | (Men- Women) ✻ Ideology | 0.30 | 0.20 | -0.08 | 0.69 | 0.26 | 345 | 1.54 | 0.124 |
| Gender ✻ D1 ✻ Ideology | (Men- Women) ✻ (Consensus - Denialist) ✻ Ideology | -0.17 | 0.28 | -0.73 | 0.39 | -0.15 | 345 | -0.60 | 0.548 |
| Gender ✻ D2 ✻ Ideology | (Men - Women) ✻ (Control - Denialist) ✻ Ideology | -0.10 | 0.29 | -0.67 | 0.48 | -0.09 | 345 | -0.33 | 0.741 |

| Parameter Estimates for simple effects of Condition | | | | | | | | | | |
| --- | --- | --- | --- | --- | --- | --- | --- | --- | --- | --- |
| **Moderator** | |  | | | **95% Confidence Intervals** | |  | | | |
| **Ideological Orientation** | **Gender** | **Effect** | **Estimate** | **SE** | **Lower** | **Upper** | **β** | **df** | **t** | **p** |
| **50-34%** | **Woman** | **Consesus - Denialist** | -0.09 | 0.20 | -0.49 | 0.31 | -0.07 | 350 | -0.43 | 0.668 |
|  | **Woman** | **Control - Denialist** | -0.10 | 0.21 | -0.51 | 0.31 | -0.08 | 350 | -0.47 | 0.639 |
|  | **Man** | **Consesus - Denialist** | -0.18 | 0.25 | -0.67 | 0.31 | -0.13 | 350 | -0.71 | 0.478 |
|  | **Man** | **Control - Denialist** | -0.29 | 0.26 | -0.80 | 0.23 | -0.22 | 350 | -1.09 | 0.275 |
| **50%** | **Woman** | **Consesus - Denialist** | -0.09 | 0.20 | -0.49 | 0.31 | -0.07 | 350 | -0.43 | 0.668 |
|  | **Woman** | **Control - Denialist** | -0.10 | 0.21 | -0.51 | 0.31 | -0.08 | 350 | -0.47 | 0.639 |
|  | **Man** | **Consesus - Denialist** | -0.18 | 0.25 | -0.67 | 0.31 | -0.13 | 350 | -0.71 | 0.478 |
|  | **Man** | **Control - Denialist** | -0.29 | 0.26 | -0.80 | 0.23 | -0.22 | 350 | -1.09 | 0.275 |
| **50+34%** | **Woman** | **Consesus - Denialist** | -0.09 | 0.20 | -0.49 | 0.31 | -0.07 | 350 | -0.43 | 0.668 |
|  | **Woman** | **Control - Denialist** | -0.10 | 0.21 | -0.51 | 0.31 | -0.08 | 350 | -0.47 | 0.639 |
|  | **Man** | **Consesus - Denialist** | -0.18 | 0.25 | -0.67 | 0.31 | -0.13 | 350 | -0.71 | 0.478 |
|  | **Man** | **Control - Denialist** | -0.29 | 0.26 | -0.80 | 0.23 | -0.22 | 350 | -1.09 | 0.275 |

# Separate analysis for subcategories of the instrument “Intention to Perform Pro-Environmental Behaviors” in Ecuador (Study 2)

*Effects of Condition, Gender, Ideological Orientation and Their Interactions on the Intention Not to Have a Pet*

| **Predictor** | ***b*** | ***HE*** | ***t*** | ***p*** | **LLCI** | **ULCI** |
| --- | --- | --- | --- | --- | --- | --- |
| **Constant** | **2.97** | **0.24** | **12.13** | **<.001** | **2.48** | **3.45** |
| D1 | -0.03 | 0.33 | -0.11 | .911 | -0.69 | 0.62 |
| D2 | -0.36 | 0.34 | -1.07 | .283 | -1.03 | 0.30 |
| Gender | -0.32 | 0.36 | -0.91 | .361 | -1.03 | 0.37 |
| Ideology | 0.39 | 0.23 | 1.69 | .092 | -0.06 | 0.86 |
| D1 X Gender | 0.05 | 0.53 | 0.10 | .919 | -0.99 | 1.10 |
| D2 X Gender | 0.64 | 0.54 | 1.18 | .236 | -0.42 | 1.70 |
| D1 X Ideology | 0.53 | 0.30 | -1.75 | .081 | -1.13 | 0.06 |
| D2 X Ideology | 0.06 | 0.32 | 0.19 | .845 | -0.57 | 0.69 |
| **Gender X Ideology** | **-0.83** | **0.31** | **-2.63** | **.009** | **-1.45** | **-0.20** |
| Left | 0.41 | 0.46 | 0.89 | .371 | -0.49 | 1.31 |
| **Right** | **-1.01** | **0.44** | **-2.29** | **.022** | **-1.88** | **-0.14** |
| **D1 X Gender X Ideology** | **1.39** | **0.45** | **3.04** | **.002** | **0.49** | **2.30** |
| Woman -Left | 0.44 | 0.39 | 1.10 | .268 | -0.34 | 1.22 |
| Woman-Right | -0.48 | 0.45 | -1.06 | .287 | -1.36 | 0.40 |
| Man-Left | -0.75 | 0.57 | -1.31 | .190 | -1.88 | 0.37 |
| Man-Right | 0.73 | 0.43 | 1.68 | .094 | -0.12 | 1.53 |
| D2​ X Gender X Ideology | -0.26 | 0.47 | -0.55 | .577 | -1.20 | 0.66 |
| Woman -Left | -0.42 | 0.43 | 0.96 | .335 | -1.28 | 0.43 |
| Woman-Right | -0.31 | 0.44 | -0.71 | .477 | -1.18 | 0.55 |
| Man-Left | 0.45 | 0.51 | 0.89 | .373 | -0.54 | 1.46 |
| Man-Right | 0.10 | 0.52 | 0.21 | .083 | -0.91 | 1.13 |

Note. LLCI = Lower level confidence interval; ULCI = upper-level confidence interval. Significant effects appear in bold. Conditional effects appear indented.

*Effects of Condition, Gender, Ideological Orientation and Their Interactions in the Intention to Modify Transportation Habits*

| **Predictor** | ***b*** | ***HE*** | ***β*** | ***t*** | ***p*** | **LLCI** | **ULCI** |
| --- | --- | --- | --- | --- | --- | --- | --- |
| **Constant** | 4.72 | 0.13 | -0.01 | 35.27 | <.001 | 4.46 | 4.99 |
| **D1** | **0.39** | **0.18** | **0.34** | **2.16** | **.031** | **0.06** | **0.75** |
| D2 | -0.15 | 0.18 | -0.13 | -0.82 | .408 | -0.52 | 0.21 |
| Gender | -0.10 | 0.19 | -0.09 | -0.55 | .583 | -0.49 | 0.27 |
| Ideology | -0.17 | 0.12 | -0.17 | -1.36 | .174 | -0.42 | 0.07 |
| D1 X Gender | -0.43 | 0.29 | -0.37 | -1.49 | .137 | -1.00 | 0.13 |
| D2 X Gender | 0.26 | 0.29 | 0.23 | 0.90 | .365 | -0.31 | 0.85 |
| D1 X Ideology | 0.07 | 0.16 | 0.07 | 0.45 | .652 | -0.25 | 0.40 |
| D2 X Ideology | 0.12 | 0.17 | 0.12 | 0.69 | .490 | -0.22 | 0.47 |
| **Gender X Ideology** | **-0.47** | **0.17** | **-0.47** | **-2.74** | **.006** | **-0.81** | **-0.13** |
| Left | 0.31 | 0.25 | -0.11 | 1.24 | .213 | -1.18 | 0.80 |
| **Right** | **-0.50** | **0.24** | **-0.24** | **-2.06** | **.039** | **-0.97** | **-0.02** |
| **D1 X Gender X Ideology** | **0.53** | **0.25** | **0.52** | **2.11** | **.035** | **0.03** | **1.02** |
| Woman -Left | 0.32 | 0.21 | 0.30 | 1.51 | .131 | -0.09 | 0.75 |
| Woman-Right | 0.45 | 0.24 | 0.40 | 1.85 | .064 | -0.02 | 0.94 |
| Man-Left | 0.58 | 0.31 | -0.38 | -1.84 | .066 | -1.19 | 0.03 |
| Man-Right | 0.46 | 0.23 | 0.40 | 1.95 | .051 | -0.00 | 0.93 |
| D2 X Gender X Ideology | 0.48 | 0.26 | 0.48 | 1.85 | .064 | -0.02 | 0.99 |
| Woman -Left | -0.26 | 0.24 | -0.20 | 1.51 | .131 | -0.09 | 0.75 |
| Woman-Right | -0.05 | 0.24 | -0.05 | -0.22 | .825 | -0.02 | 0.94 |
| Man-Left | -0.42 | 0.28 | -0.25 | -1.58 | .128 | -0.97 | 0.12 |
| **Man-Right** | **0.61** | **0.23** | **0.53** | **2.15** | **.032** | **0.05** | **1.17** |

*Note*. LLCI = Lower level confidence interval; ULCI = upper-level confidence interval. . Significant effects appear in bold. Conditional effects appear indented.

*Effects of Condition, Gender, Ideological Orientation and Their Interactions in the Intention to reduce and recycle*

| **Predictor** | ***b*** | ***HE*** | ***β*** | ***t*** | ***p*** | **LLCI** | **ULCI** |
| --- | --- | --- | --- | --- | --- | --- | --- |
| (Intercept) | 5.85 | 0.12 | 0.04 | 48.92 | <.001 | 5.61 | 6.08 |
| Gender | -0.35 | 0.18 | -0.34 | -1.99 | 0.047 | -0.70 | -0.00 |
| D1 | 0.22 | 0.16 | 0.22 | 1.35 | 0.179 | -0.10 | 0.54 |
| D2 | 0.11 | 0.17 | 0.11 | 0.68 | 0.496 | -0.21 | 0.44 |
| Ideological Orientation | -0.13 | 0.11 | -0.15 | -1.17 | 0.241 | -0.36 | 0.09 |
| Gender ✻ Condition1 | -0.20 | 0.26 | -0.20 | -0.79 | 0.433 | -0.71 | 0.31 |
| Gender ✻ Condition2 | 0.22 | 0.26 | 0.22 | 0.85 | 0.397 | -0.30 | 0.74 |
| Gender ✻ Ideological Orientation | -0.28 | 0.15 | -0.31 | -1.81 | 0.072 | -0.58 | 0.02 |
| D1 ✻ Ideological Orientation | 0.05 | 0.15 | 0.05 | 0.31 | 0.756 | -0.25 | 0.34 |
| D2 ✻ Ideological Orientation | 0.13 | 0.16 | 0.14 | 0.80 | 0.424 | -0.18 | 0.44 |
| Gender ✻ D1 ✻ Ideological Orientation | 0.31 | 0.22 | 0.34 | 1.38 | 0.170 | -0.13 | 0.75 |
| Gender ✻ D2✻ Ideological Orientation | 0.18 | 0.23 | 0.20 | 0.77 | 0.442 | -0.28 | 0.63 |

*Note*. LLCI = Lower level confidence interval; ULCI = upper-level confidence interval. . Significant effects appear in bold. Conditional effects appear indented.

*Effects of Condition, Gender, Ideological Orientation and Their Interactions in the Intention to change the diet*

| **Predictor** | ***b*** | ***HE*** | ***β*** | ***t*** | ***p*** | **LLCI** | **ULCI** |
| --- | --- | --- | --- | --- | --- | --- | --- |
| (Intercept) | 4.27 | 0.17 | 0.19 | 24.42 | <.001 | 3.92 | 4.61 |
| Gender1 | -0.60 | 0.26 | -0.40 | -2.32 | 0.021 | -1.10 | -0.09 |
| D1 | 0.16 | 0.24 | 0.11 | 0.68 | 0.497 | -0.31 | 0.63 |
| D2 | -0.37 | 0.24 | -0.25 | -1.51 | 0.131 | -0.85 | 0.11 |
| Ideological Orientation | -0.20 | 0.17 | -0.15 | -1.17 | 0.241 | -0.53 | 0.13 |
| Gender ✻ D1 | -0.13 | 0.38 | -0.08 | -0.33 | 0.739 | -0.87 | 0.62 |
| Gender ✻ D2 | 0.34 | 0.39 | 0.23 | 0.89 | 0.375 | -0.42 | 1.10 |
| Gender✻ Ideological Orientation | -0.33 | 0.23 | -0.25 | -1.45 | 0.147 | -0.77 | 0.12 |
| D1 ✻ Ideological Orientation | 0.01 | 0.22 | 0.01 | 0.05 | 0.962 | -0.42 | 0.44 |
| D2 ✻ Ideological Orientation | 0.14 | 0.23 | 0.11 | 0.62 | 0.533 | -0.31 | 0.60 |
| Gender ✻ D1 ✻ Ideological Orientation | 0.27 | 0.33 | 0.21 | 0.83 | 0.407 | -0.37 | 0.92 |
| Gender ✻ D2 ✻ Ideological Orientation | 0.23 | 0.34 | 0.18 | 0.67 | 0.500 | -0.44 | 0.90 |

*Note*. LLCI = Lower level confidence interval; ULCI = upper-level confidence interval. . Significant effects appear in bold. Conditional effects appear indented.

# Study 2 Ecuador: Alternative analyses comparing the consensus condition and the control condition

**Intention to Perform Pro-Environmental Behaviors**

| Model Fit | | | | | |
| --- | --- | --- | --- | --- | --- |
| **R²** | **Adj. R²** | **df** | **df (res)** | **F** | **p** |
| 0.17 | 0.14 | 11 | 353 | 6.35 | <.001 |

| Parameter Estimates (Coefficients) | | | | | | | | | |
| --- | --- | --- | --- | --- | --- | --- | --- | --- | --- |
|  | | | | **95% Confidence Intervals** | |  | | | |
| **Names** | **Effect** | **Estimate** | **SE** | **Lower** | **Upper** | **β** | **df** | **t** | **p** |
| (Intercept) | (Intercept) | 5.17 | 0.10 | 4.98 | 5.36 | 0.37 | 353 | 53.77 | <.001 |
| Gender | Men - Women | -0.58 | 0.16 | -0.90 | -0.25 | -0.63 | 353 | -3.51 | <.001 |
| D1 | 1 Denialist - 2 Consensus | -0.23 | 0.14 | -0.51 | 0.04 | -0.26 | 353 | -1.67 | 0.096 |
| D2 | 0 Control - 2 Consensus | -0.35 | 0.14 | -0.63 | -0.08 | -0.39 | 353 | -2.55 | 0.011 |
| Ideology | Ideology | -0.12 | 0.08 | -0.28 | 0.04 | -0.15 | 353 | -1.47 | 0.143 |
| Gender ✻ D1 | (Men - Women) ✻ (1 Denialist - 2 Consensus) | 0.23 | 0.22 | -0.21 | 0.67 | 0.25 | 353 | 1.02 | 0.309 |
| Gender ✻ D2 | (Men - Women) ✻ (0 Control - 2 Consensus) | 0.52 | 0.24 | 0.06 | 0.99 | 0.57 | 353 | 2.21 | 0.027 |
| Gender ✻ Ideology | (Men - Women) ✻ Ideology | 0.05 | 0.14 | -0.22 | 0.33 | 0.06 | 353 | 0.36 | 0.716 |
| D1 ✻ Ideology | (1 Denialist - 2 Consensus) ✻ Ideology | -0.01 | 0.13 | -0.26 | 0.25 | -0.01 | 353 | -0.05 | 0.964 |
| D2 ✻ Ideology | (0 Control - 2 Consensus) ✻ Ideology | 0.12 | 0.12 | -0.12 | 0.36 | 0.15 | 353 | 0.96 | 0.335 |
| Gender ✻ D1 ✻ Ideology | (Men - Women) ✻ (1 Denialist - 2 Consensus) ✻ Ideology | -0.43 | 0.19 | -0.81 | -0.05 | -0.54 | 353 | -2.24 | 0.026 |
| Gender ✻ D2 ✻ Ideology | (Men - Women) ✻ (0 Control - 2 Consensus) ✻ Ideology | -0.19 | 0.20 | -0.59 | 0.21 | -0.24 | 353 | -0.92 | 0.360 |

| ANOVA for Simple Effects of Condition | | | | | | |
| --- | --- | --- | --- | --- | --- | --- |
| **Moderator** | |  | | | | |
| **Ideology** | **Gender** | **F** | **Num df** | **Den df** | **p** | **η²p** |
| **50-34%** | **Women** | 3.82 | 2 | 353 | 0.023 | 0.02 |
|  | **Men** | 0.87 | 2 | 353 | 0.419 | 0.00 |
| **50%** | **Women** | 3.74 | 2 | 353 | 0.025 | 0.02 |
|  | **Men** | 0.45 | 2 | 353 | 0.640 | 0.00 |
| **50+34%** | **Women** | 1.25 | 2 | 353 | 0.288 | 0.01 |
|  | **Men** | 3.27 | 2 | 353 | 0.039 | 0.02 |

| Parameter Estimates for simple effects of Condition | | | | | | | | | | |
| --- | --- | --- | --- | --- | --- | --- | --- | --- | --- | --- |
| **Moderator** | |  | | | **95% Confidence Intervals** | |  | | | |
| **Ideology** | **Gender** | **Effect** | **Estimate** | **SE** | **Lower** | **Upper** | **β** | **df** | **t** | **p** |
| **50-34%** | **Women** | **Consenso - Negacionista** | 0.23 | 0.15 | -0.07 | 0.53 | 0.25 | 353 | 1.51 | 0.132 |
|  | **Women** | **Control - Negacionista** | -0.20 | 0.17 | -0.53 | 0.13 | -0.22 | 353 | -1.22 | 0.224 |
|  | **Men** | **Consenso - Negacionista** | -0.29 | 0.22 | -0.72 | 0.14 | -0.31 | 353 | -1.31 | 0.192 |
|  | **Men** | **Control - Negacionista** | -0.07 | 0.20 | -0.46 | 0.32 | -0.08 | 353 | -0.36 | 0.719 |
| **50%** | **Women** | **Consenso - Negacionista** | 0.23 | 0.14 | -0.04 | 0.51 | 0.26 | 353 | 1.68 | 0.093 |
|  | **Women** | **Control - Negacionista** | -0.14 | 0.14 | -0.42 | 0.14 | -0.15 | 353 | -0.97 | 0.331 |
|  | **Men** | **Consenso - Negacionista** | -0.07 | 0.18 | -0.42 | 0.29 | -0.07 | 353 | -0.38 | 0.707 |
|  | **Men** | **Control - Negacionista** | 0.11 | 0.18 | -0.24 | 0.46 | 0.12 | 353 | 0.64 | 0.522 |
| **50+34%** | **Women** | **Consenso - Negacionista** | 0.24 | 0.19 | -0.13 | 0.61 | 0.26 | 353 | 1.27 | 0.206 |
|  | **Women** | **Control - Negacionista** | -0.01 | 0.19 | -0.38 | 0.35 | -0.02 | 353 | -0.08 | 0.937 |
|  | **Men** | **Consenso - Negacionista** | 0.37 | 0.18 | 0.01 | 0.73 | 0.40 | 353 | 2.02 | 0.044 |
|  | **Men** | **Control - Negacionista** | 0.48 | 0.22 | 0.05 | 0.91 | 0.53 | 353 | 2.21 | 0.028 |

**Willingness to Participate in Collective Action Against Climate Change**

**Model Results**

| Model Fit | | | | | |
| --- | --- | --- | --- | --- | --- |
| **R²** | **Adj. R²** | **df** | **df (res)** | **F** | **p** |
| 0.10 | 0.07 | 11 | 350 | 3.40 | <.001 |

| Parameter Estimates (Coefficients) | | | | | | | | | |
| --- | --- | --- | --- | --- | --- | --- | --- | --- | --- |
|  | | | | **95% Confidence Intervals** | |  | | | |
| **Names** | **Effect** | **Estimate** | **SE** | **Lower** | **Upper** | **β** | **df** | **t** | **p** |
| (Intercept) | (Intercept) | 4.34 | 0.15 | 4.05 | 4.62 | 0.11 | 350 | 29.47 | <.001 |
| Gender | Men - Women | -0.34 | 0.25 | -0.84 | 0.15 | -0.26 | 350 | -1.36 | 0.176 |
| D1 | 1 Denialist - 2 Consensus | 0.17 | 0.22 | -0.25 | 0.59 | 0.13 | 350 | 0.78 | 0.435 |
| D2 | 0 Control - 2 Consensus | -0.13 | 0.21 | -0.55 | 0.29 | -0.10 | 350 | -0.62 | 0.536 |
| Ideology | Ideology | -0.18 | 0.13 | -0.43 | 0.07 | -0.16 | 350 | -1.44 | 0.150 |
| Gender ✻ D1 | (Men - Women) ✻ (1 Denialist - 2 Consensus) | -0.18 | 0.34 | -0.85 | 0.49 | -0.13 | 350 | -0.53 | 0.600 |
| Gender ✻ D2 | (Men - Women) ✻ (0 Control - 2 Consensus) | 0.05 | 0.37 | -0.67 | 0.77 | 0.04 | 350 | 0.15 | 0.884 |
| Gender ✻ Ideology | (Men - Women) ✻ Ideology | -0.01 | 0.21 | -0.43 | 0.41 | -0.01 | 350 | -0.05 | 0.963 |
| D1 ✻ Ideology | (1 Denialist - 2 Consensus) ✻ Ideology | -0.07 | 0.20 | -0.46 | 0.32 | -0.06 | 350 | -0.36 | 0.722 |
| D2 ✻ Ideology | (0 Control - 2 Consensus) ✻ Ideology | 0.00 | 0.19 | -0.37 | 0.38 | 0.00 | 350 | 0.02 | 0.985 |
| Gender ✻ D1 ✻ Ideology | (Men - Women) ✻ (1 Denialist - 2 Consensus) ✻ Ideology | -0.27 | 0.30 | -0.85 | 0.32 | -0.23 | 350 | -0.90 | 0.369 |
| Gender ✻ D2 ✻ Ideology | (Men - Women) ✻ (0 Control - 2 Consensus) ✻ Ideology | 0.02 | 0.32 | -0.60 | 0.64 | 0.02 | 350 | 0.06 | 0.952 |

| ANOVA for Simple Effects of Condition | | | | | | |
| --- | --- | --- | --- | --- | --- | --- |
| **Moderator** | |  | | | | |
| **Ideology** | **Gender** | **F** | **Num df** | **Den df** | **p** | **η²p** |
| **50-34%** | **Women** | 0.97 | 2 | 350 | 0.380 | 0.01 |
|  | **Men** | 0.53 | 2 | 350 | 0.588 | 0.00 |
| **50%** | **Women** | 1.01 | 2 | 350 | 0.364 | 0.01 |
|  | **Men** | 0.11 | 2 | 350 | 0.897 | 0.00 |
| **50+34%** | **Women** | 0.36 | 2 | 350 | 0.697 | 0.00 |
|  | **Men** | 0.58 | 2 | 350 | 0.558 | 0.00 |

| Parameter Estimates for simple effects of Condition | | | | | | | | | | |
| --- | --- | --- | --- | --- | --- | --- | --- | --- | --- | --- |
| **Moderator** | |  | | | **95% Confidence Intervals** | |  | | | |
| **Ideology** | **Gender** | **Effect** | **Estimate** | **SE** | **Lower** | **Upper** | **β** | **df** | **t** | **p** |
| **50-34%** | **Women** | **Consenso - Negacionista** | -0.22 | 0.23 | -0.68 | 0.24 | -0.16 | 350 | -0.92 | 0.357 |
|  | **Women** | **Control - Negacionista** | -0.35 | 0.26 | -0.85 | 0.15 | -0.26 | 350 | -1.37 | 0.172 |
|  | **Men** | **Consenso - Negacionista** | -0.21 | 0.34 | -0.88 | 0.45 | -0.16 | 350 | -0.63 | 0.527 |
|  | **Men** | **Control - Negacionista** | -0.31 | 0.31 | -0.91 | 0.30 | -0.23 | 350 | -1.00 | 0.317 |
| **50%** | **Women** | **Consenso - Negacionista** | -0.18 | 0.21 | -0.60 | 0.24 | -0.13 | 350 | -0.85 | 0.396 |
|  | **Women** | **Control - Negacionista** | -0.31 | 0.22 | -0.75 | 0.12 | -0.23 | 350 | -1.42 | 0.158 |
|  | **Men** | **Consenso - Negacionista** | -0.05 | 0.28 | -0.60 | 0.50 | -0.03 | 350 | -0.17 | 0.868 |
|  | **Men** | **Control - Negacionista** | -0.13 | 0.28 | -0.67 | 0.41 | -0.10 | 350 | -0.47 | 0.642 |
| **50+34%** | **Women** | **Consenso - Negacionista** | -0.11 | 0.29 | -0.68 | 0.46 | -0.08 | 350 | -0.38 | 0.703 |
|  | **Women** | **Control - Negacionista** | -0.24 | 0.28 | -0.80 | 0.32 | -0.18 | 350 | -0.84 | 0.400 |
|  | **Men** | **Consenso - Negacionista** | 0.29 | 0.28 | -0.26 | 0.84 | 0.22 | 350 | 1.03 | 0.303 |
|  | **Men** | **Control - Negacionista** | 0.23 | 0.34 | -0.44 | 0.90 | 0.17 | 350 | 0.68 | 0.499 |

**Attitudes Towards Pro-Environmental Behaviors**

**Model Results**

| Model Fit | | | | | |
| --- | --- | --- | --- | --- | --- |
| **R²** | **Adj. R²** | **df** | **df (res)** | **F** | **p** |
| 0.09 | 0.06 | 11 | 353 | 3.17 | <.001 |

| Parameter Estimates (Coefficients) | | | | | | | | | |
| --- | --- | --- | --- | --- | --- | --- | --- | --- | --- |
|  | | | | **95% Confidence Intervals** | |  | | | |
| **Names** | **Effect** | **Estimate** | **SE** | **Lower** | **Upper** | **β** | **df** | **t** | **p** |
| (Intercept) | (Intercept) | 4.81 | 0.13 | 4.55 | 5.08 | 0.10 | 353 | 35.98 | <.001 |
| Gender | Men - Women | -0.42 | 0.23 | -0.87 | 0.03 | -0.34 | 353 | -1.82 | 0.070 |
| D1 | 1 Denialist - 2 Consensus | -0.05 | 0.20 | -0.44 | 0.33 | -0.04 | 353 | -0.27 | 0.790 |
| D2 | 0 Control - 2 Consensus | -0.02 | 0.19 | -0.40 | 0.36 | -0.02 | 353 | -0.11 | 0.912 |
| Ideology | Ideology | -0.16 | 0.11 | -0.39 | 0.06 | -0.15 | 353 | -1.43 | 0.155 |
| Gender ✻ D1 | (Men - Women) ✻ (1 Denialist - 2 Consensus) | 0.10 | 0.31 | -0.51 | 0.71 | 0.08 | 353 | 0.33 | 0.741 |
| Gender1 ✻ D2 | (Men - Women) ✻ (0 Control - 2 Consensus) | 0.49 | 0.33 | -0.16 | 1.14 | 0.40 | 353 | 1.49 | 0.137 |
| Gender ✻ Ideology | (Men - Women) ✻ Ideology | 0.20 | 0.20 | -0.18 | 0.59 | 0.19 | 353 | 1.05 | 0.294 |
| D1 ✻ Ideology | (1 Denialist - 2 Consensus) ✻ Ideology | -0.01 | 0.18 | -0.36 | 0.34 | -0.01 | 353 | -0.06 | 0.956 |
| D2 ✻ Ideology | (0 Control - 2 Consensus) ✻ Ideology | 0.26 | 0.17 | -0.08 | 0.60 | 0.25 | 353 | 1.52 | 0.129 |
| Gender ✻ D1 ✻ Ideology | (Men - Women) ✻ (1 Denialist - 2 Consensus) ✻ Ideology | -0.56 | 0.27 | -1.08 | -0.03 | -0.52 | 353 | -2.07 | 0.039 |
| Gender ✻ D2 ✻ Ideology | (Men - Women) ✻ (0 Control - 2 Consensus) ✻ Ideology | -0.44 | 0.28 | -1.00 | 0.12 | -0.42 | 353 | -1.56 | 0.120 |

| ANOVA for Simple Effects of Condition | | | | | | |
| --- | --- | --- | --- | --- | --- | --- |
| **Moderator** | |  | | | | |
| **Ideology** | **Gender** | **F** | **Num df** | **Den df** | **p** | **η²p** |
| **50-34%** | **Women** | 0.42 | 2 | 353 | 0.655 | 0.00 |
|  | **Men** | 1.81 | 2 | 353 | 0.165 | 0.01 |
| **50%** | **Women** | 0.07 | 2 | 353 | 0.936 | 0.00 |
|  | **Men** | 1.83 | 2 | 353 | 0.161 | 0.01 |
| **50+34%** | **Women** | 0.57 | 2 | 353 | 0.564 | 0.00 |
|  | **Men** | 3.26 | 2 | 353 | 0.040 | 0.02 |

| Model Info | | |
| --- | --- | --- |
| **Info** |  |  |
| Model Type | Linear Model | OLS Model for continuous y |
| Model | lm | `Attitudes Towards Pro-Environmental Behaviors` ~ 1 + Gender + Condition + Ideology + Gender:Condition + Condition:Ideology + Gender:Ideology + Gender:Condition:Ideology |
| Distribution | Gaussian | Normal distribution of residuals |
| Omnibus Tests | F |  |
| Sample size | 365 |  |
| Converged | yes |  |
| Y transform | none |  |
| C.I. method | Wald |  |
| Nota. All covariates are centered to the mean | | |

| Model Fit | | | | | |
| --- | --- | --- | --- | --- | --- |
| **R²** | **Adj. R²** | **df** | **df (res)** | **F** | **p** |
| 0.09 | 0.06 | 11 | 353 | 3.17 | <.001 |

| ANOVA Omnibus tests | | | | | |
| --- | --- | --- | --- | --- | --- |
|  | **SS** | **df** | **F** | **p** | **η²p** |
| **Model** | 48.28 | 11 | 3.17 | <.001 | 0.09 |
| **Gender** | 3.07 | 1 | 2.22 | 0.137 | 0.01 |
| **Condition** | 0.10 | 2 | 0.04 | 0.965 | 0.00 |
| **Ideology** | 2.21 | 1 | 1.60 | 0.207 | 0.00 |
| **Gender ✻ Condition** | 3.43 | 2 | 1.24 | 0.291 | 0.01 |
| **Condition ✻ Ideology** | 4.05 | 2 | 1.46 | 0.233 | 0.01 |
| **Gender ✻ Ideology** | 5.04 | 1 | 3.64 | 0.057 | 0.01 |
| **Gender ✻ Condition ✻ Ideology** | 6.46 | 2 | 2.33 | 0.098 | 0.01 |
| **Residuals** | 488.29 | 353 |  |  |  |
| **Total** | 536.58 | 364 |  |  |  |

| Parameter Estimates (Coefficients) | | | | | | | | | |
| --- | --- | --- | --- | --- | --- | --- | --- | --- | --- |
|  | | | | **95% Confidence Intervals** | |  | | | |
| **Names** | **Effect** | **Estimate** | **SE** | **Lower** | **Upper** | **β** | **df** | **t** | **p** |
| (Intercept) | (Intercept) | 4.76 | 0.14 | 4.48 | 5.04 | 0.05 | 353 | 33.32 | <.001 |
| Gender1 | Men - Women | -0.31 | 0.21 | -0.73 | 0.10 | -0.26 | 353 | -1.49 | 0.137 |
| Condition1 | Consenso - Negacionista | 0.05 | 0.20 | -0.33 | 0.44 | 0.04 | 353 | 0.27 | 0.790 |
| Condition2 | Control - Negacionista | 0.03 | 0.20 | -0.36 | 0.42 | 0.03 | 353 | 0.15 | 0.878 |
| Ideology | Ideology | -0.17 | 0.14 | -0.44 | 0.10 | -0.16 | 353 | -1.26 | 0.207 |
| Gender1 ✻ Condition1 | (Men - Women) ✻ (Consenso - Negacionista) | -0.10 | 0.31 | -0.71 | 0.51 | -0.08 | 353 | -0.33 | 0.741 |
| Gender1 ✻ Condition2 | (Men - Women) ✻ (Control - Negacionista) | 0.39 | 0.32 | -0.23 | 1.01 | 0.32 | 353 | 1.23 | 0.221 |
| Condition1 ✻ Ideology | (Consenso - Negacionista) ✻ Ideology | 0.01 | 0.18 | -0.34 | 0.36 | 0.01 | 353 | 0.06 | 0.956 |
| Condition2 ✻ Ideology | (Control - Negacionista) ✻ Ideology | 0.27 | 0.19 | -0.10 | 0.64 | 0.26 | 353 | 1.45 | 0.148 |
| Gender1 ✻ Ideology | (Men - Women) ✻ Ideology | -0.35 | 0.18 | -0.71 | 0.01 | -0.33 | 353 | -1.91 | 0.057 |
| Gender1 ✻ Condition1 ✻ Ideology | (Men - Women) ✻ (Consenso - Negacionista) ✻ Ideology | 0.56 | 0.27 | 0.03 | 1.08 | 0.52 | 353 | 2.07 | 0.039 |
| Gender1 ✻ Condition2 ✻ Ideology | (Men - Women) ✻ (Control - Negacionista) ✻ Ideology | 0.11 | 0.28 | -0.43 | 0.66 | 0.11 | 353 | 0.41 | 0.685 |

| ANOVA for Simple Effects of Condition | | | | | | |
| --- | --- | --- | --- | --- | --- | --- |
| **Moderator** | |  | | | | |
| **Ideology** | **Gender** | **F** | **Num df** | **Den df** | **p** | **η²p** |
| **50-34%** | **Women** | 0.42 | 2 | 353 | 0.655 | 0.00 |
|  | **Men** | 1.81 | 2 | 353 | 0.165 | 0.01 |
| **50%** | **Women** | 0.07 | 2 | 353 | 0.936 | 0.00 |
|  | **Men** | 1.83 | 2 | 353 | 0.161 | 0.01 |
| **50+34%** | **Women** | 0.57 | 2 | 353 | 0.564 | 0.00 |
|  | **Men** | 3.26 | 2 | 353 | 0.040 | 0.02 |

| Parameter Estimates for simple effects of Condition | | | | | | | | | | |
| --- | --- | --- | --- | --- | --- | --- | --- | --- | --- | --- |
| **Moderator** | |  | | | **95% Confidence Intervals** | |  | | | |
| **Ideology** | **Gender** | **Effect** | **Estimate** | **SE** | **Lower** | **Upper** | **β** | **df** | **t** | **p** |
| **50-34%** | **Women** | **Consenso - Negacionista** | 0.05 | 0.21 | -0.37 | 0.46 | 0.04 | 353 | 0.21 | 0.831 |
|  | **Women** | **Control - Negacionista** | -0.15 | 0.23 | -0.61 | 0.30 | -0.13 | 353 | -0.66 | 0.510 |
|  | **Men** | **Consenso - Negacionista** | -0.43 | 0.31 | -1.03 | 0.17 | -0.36 | 353 | -1.41 | 0.159 |
|  | **Men** | **Control - Negacionista** | 0.16 | 0.28 | -0.38 | 0.70 | 0.13 | 353 | 0.58 | 0.565 |
| **50%** | **Women** | **Consenso - Negacionista** | 0.05 | 0.19 | -0.33 | 0.43 | 0.04 | 353 | 0.26 | 0.795 |
|  | **Women** | **Control - Negacionista** | -0.02 | 0.20 | -0.41 | 0.38 | -0.01 | 353 | -0.08 | 0.934 |
|  | **Men** | **Consenso - Negacionista** | -0.15 | 0.25 | -0.64 | 0.35 | -0.12 | 353 | -0.59 | 0.557 |
|  | **Men** | **Control - Negacionista** | 0.35 | 0.25 | -0.13 | 0.84 | 0.29 | 353 | 1.43 | 0.154 |
| **50+34%** | **Women** | **Consenso - Negacionista** | 0.06 | 0.26 | -0.46 | 0.58 | 0.05 | 353 | 0.23 | 0.819 |
|  | **Women** | **Control - Negacionista** | 0.26 | 0.26 | -0.25 | 0.76 | 0.21 | 353 | 1.00 | 0.320 |
|  | **Men** | **Consenso - Negacionista** | 0.42 | 0.25 | -0.08 | 0.92 | 0.34 | 353 | 1.65 | 0.101 |
|  | **Men** | **Control - Negacionista** | 0.74 | 0.30 | 0.14 | 1.34 | 0.61 | 353 | 2.43 | 0.016 |

**Perceived Behavioral Control over Pro-Environmental Behaviors*.***

**Model Results**

| Model Fit | | | | | |
| --- | --- | --- | --- | --- | --- |
| **R²** | **Adj. R²** | **df** | **df (res)** | **F** | **p** |
| 0.06 | 0.03 | 11 | 353 | 1.87 | 0.042 |

| Parameter Estimates (Coefficients) | | | | | | | | | |
| --- | --- | --- | --- | --- | --- | --- | --- | --- | --- |
|  | | | | **95% Confidence Intervals** | |  | | | |
| **Names** | **Effect** | **Estimate** | **SE** | **Lower** | **Upper** | **β** | **df** | **t** | **p** |
| (Intercept) | (Intercept) | 4.51 | 0.18 | 4.16 | 4.85 | -0.04 | 353 | 25.61 | <.001 |
| Gender | Men - Women | -0.01 | 0.30 | -0.60 | 0.58 | -0.01 | 353 | -0.03 | 0.977 |
| D1 | 1 Denialist - 2 Consensus | -0.01 | 0.26 | -0.52 | 0.49 | -0.01 | 353 | -0.06 | 0.954 |
| D2 | 0 Control - 2 Consensus | 0.36 | 0.25 | -0.14 | 0.85 | 0.23 | 353 | 1.40 | 0.162 |
| Ideology | Ideology | 0.09 | 0.15 | -0.21 | 0.38 | 0.06 | 353 | 0.58 | 0.562 |
| Gender ✻ D2 | (Men - Women) ✻ (1 Denialist - 2 Consensus) | -0.30 | 0.41 | -1.10 | 0.50 | -0.19 | 353 | -0.74 | 0.462 |
| Gender ✻ D2 | (Men - Women) ✻ (0 Control - 2 Consensus) | 0.22 | 0.43 | -0.63 | 1.07 | 0.14 | 353 | 0.51 | 0.611 |
| Gender ✻ Ideology | (Men - Women) ✻ Ideology | 0.01 | 0.26 | -0.50 | 0.51 | 0.00 | 353 | 0.02 | 0.983 |
| D1 ✻ Ideology | (1 Denialist - 2 Consensus) ✻ Ideology | -0.20 | 0.24 | -0.66 | 0.27 | -0.14 | 353 | -0.84 | 0.403 |
| D2 ✻ Ideology | (0 Control - 2 Consensus) ✻ Ideology | 0.08 | 0.23 | -0.37 | 0.53 | 0.06 | 353 | 0.35 | 0.728 |
| Gender ✻ D1 ✻ Ideology | (Men - Women) ✻ (1 Denialist - 2 Consensus) ✻ Ideology | -0.29 | 0.35 | -0.98 | 0.41 | -0.21 | 353 | -0.81 | 0.417 |
| Gender ✻ D2 ✻ Ideology | (Men - Women) ✻ (0 Control - 2 Consensus) ✻ Ideology | -0.19 | 0.37 | -0.92 | 0.55 | -0.14 | 353 | -0.50 | 0.614 |

| ANOVA for Simple Effects of Condition | | | | | | |
| --- | --- | --- | --- | --- | --- | --- |
| **Moderator** | |  | | | | |
| **Ideology** | **Gender** | **F** | **Num df** | **Den df** | **p** | **η²p** |
| **50-34%** | **Women** | 0.54 | 2 | 353 | 0.581 | 0.00 |
|  | **Men** | 1.90 | 2 | 353 | 0.151 | 0.01 |
| **50%** | **Women** | 1.09 | 2 | 353 | 0.338 | 0.01 |
|  | **Men** | 3.32 | 2 | 353 | 0.037 | 0.02 |
| **50+34%** | **Women** | 1.72 | 2 | 353 | 0.180 | 0.01 |
|  | **Men** | 5.11 | 2 | 353 | 0.006 | 0.03 |

| Parameter Estimates for simple effects of Condition | | | | | | | | | | |
| --- | --- | --- | --- | --- | --- | --- | --- | --- | --- | --- |
| **Moderator** | |  | | | **95% Confidence Intervals** | |  | | | |
| **Ideology** | **Gender** | **Effect** | **Estimate** | **SE** | **Lower** | **Upper** | **β** | **df** | **t** | **p** |
| **50-34%** | **Women** | **Consenso - Negacionista** | -0.12 | 0.28 | -0.67 | 0.43 | -0.08 | 353 | -0.42 | 0.675 |
|  | **Women** | **Control - Negacionista** | 0.18 | 0.31 | -0.42 | 0.79 | 0.12 | 353 | 0.61 | 0.545 |
|  | **Men** | **Consenso - Negacionista** | -0.01 | 0.40 | -0.80 | 0.78 | -0.01 | 353 | -0.02 | 0.980 |
|  | **Men** | **Control - Negacionista** | 0.64 | 0.36 | -0.07 | 1.35 | 0.41 | 353 | 1.77 | 0.078 |
| **50%** | **Women** | **Consenso - Negacionista** | -0.02 | 0.25 | -0.52 | 0.48 | -0.01 | 353 | -0.08 | 0.940 |
|  | **Women** | **Control - Negacionista** | 0.32 | 0.26 | -0.20 | 0.84 | 0.21 | 353 | 1.22 | 0.222 |
|  | **Men** | **Consenso - Negacionista** | 0.23 | 0.33 | -0.42 | 0.88 | 0.15 | 353 | 0.70 | 0.485 |
|  | **Men** | **Control - Negacionista** | 0.83 | 0.32 | 0.19 | 1.46 | 0.53 | 353 | 2.55 | 0.011 |
| **50+34%** | **Women** | **Consenso - Negacionista** | 0.18 | 0.35 | -0.50 | 0.86 | 0.11 | 353 | 0.51 | 0.609 |
|  | **Women** | **Control - Negacionista** | 0.60 | 0.34 | -0.07 | 1.27 | 0.38 | 353 | 1.77 | 0.078 |
|  | **Men** | **Consenso - Negacionista** | 0.72 | 0.33 | 0.06 | 1.37 | 0.46 | 353 | 2.14 | 0.033 |
|  | **Men** | **Control - Negacionista** | 1.20 | 0.40 | 0.41 | 1.99 | 0.77 | 353 | 3.00 | 0.003 |
